# Cancer Vaccines for Triple-Negative Breast Cancer: A Systematic Review

**DOI:** 10.3390/vaccines11010146

**Published:** 2023-01-09

**Authors:** Mina Hosseini, Simin Seyedpour, Behzad Khodaei, Amir-Hossein Loghman, Nasrin Seyedpour, Mohammad-Hossein Yazdi, Nima Rezaei

**Affiliations:** 1 Department of Pharmaceutical Biotechnology, School of Pharmacy, Tehran University of Medical Sciences, Tehran P.O. Box 141556451, Iran; 2 School of Medicine, Tehran University of Medical Sciences, Tehran P.O. Box 1417613151, Iran; 3 Nanomedicine Research Association (NRA), Universal Scientific Education and Research Network (USERN), Tehran P.O. Box 1416634793, Iran; 4 School of Medicine, Kashan University of Medical Sciences, Kashan P.O. Box 8715988141, Iran; 5 Department of Medical Physics and Biomedical Engineering, Tehran University of Medical Sciences, Tehran P.O. Box 141556559, Iran; 6 Biotechnology Research Center, Tehran University of Medical Sciences, Tehran P.O. Box 141556451, Iran; 7 Recombinant Vaccine Research Center, Tehran University of Medical Sciences, Tehran P.O. Box 141556451, Iran; 8 Research Center for Immunodeficiencies (RCID), Children’s Medical Center, Tehran University of Medical Sciences, Qarib Street, Tehran P.O. Box 14185863, Iran; 9 Department of Immunology, School of Medicine, Tehran University of Medical Sciences, Tehran P.O. Box 141761351, Iran

**Keywords:** triple-negative breast cancer, immunotherapy, cancer vaccines, systematic review

## Abstract

Triple-negative breast cancer (TNBC) is the subtype of breast cancer with the poorest outcomes, and is associated with a high risk of relapse and metastasis. The treatment choices for this malignancy have been confined to conventional chemotherapeutic agents, due to a lack of expression of the canonical molecular targets. Immunotherapy has been recently changing the treatment paradigm for many types of tumors, and the approach of evoking active immune responses in the milieu of breast tumors through cancer vaccines has been introduced as one of the most novel immunotherapeutic approaches. Accordingly, a number of vaccines for the treatment or prevention of recurrence have been developed and are currently being studied in TNBC patients, while none have yet received any approvals. To elucidate the efficacy and safety of these vaccines, we performed a systematic review of the available literature on the topic. After searching the PubMed, Scopus, Web of Science, Embase, Cochrane CENTRAL, and Google Scholar databases, a total of 5701 results were obtained, from which 42 clinical studies were eventually included based on the predefined criteria. The overall quality of the included studies was acceptable. However, due to a lack of reporting outcomes of survival or progression in some studies (which were presented as conference abstracts) as well as the heterogeneity of the reported outcomes and study designs, we were not able to carry out a meta-analysis. A total of 32 different vaccines have so far been evaluated in TNBC patients, with the majority belonging to the peptide-based vaccine type. The other vaccines were in the cell or nucleic acid (RNA/DNA)-based categories. Most vaccines proved to be safe with low-grade, local adverse events and could efficiently evoke cellular immune responses; however, most trials were not able to demonstrate significant improvements in clinical indices of efficacy. This is in part due to the limited number of randomized studies, as well as the limited TNBC population of each trial. However, due to the encouraging results of the currently published trials, we anticipate that this strategy could show its potential through larger, phase III randomized studies in the near future.

## 1. Introduction

Breast cancer is the most common cancer in females, with increasing annual incidence and mortality rates worldwide [1]. Triple-negative breast cancer (TNBC) is a subtype of breast cancer comprising about 15% of the cases, and is defined by the lack of estrogen and progesterone receptors as well as HER2 overexpression. TNBC mainly presents with large-sized, aggressive tumors, and is known to have the worst survival outcomes among breast cancer subtypes. Most TNBC patients, especially those with a metastatic disease, experience poor prognoses, as the risk of recurrence in case of residual disease after treatment is relatively high [2,3]. A lack of the canonical molecular targets as well as the heterogeneity of TNBC tumors are among the top factors contributing to its unmet treatment challenge, and thus, chemotherapy has remained the most viable option for early and advanced stage TNBC [4]. Fortunately, recent developments have shed light on our understanding of the disease, and plenty of novel agents are finding their way into the routine clinical practice for TNBC patients, including state-of-the-art targeted and immunotherapeutic agents.

With the development of molecular targeted therapies, poly (ADP-ribose) polymerase (PARP) inhibitors, immune checkpoint blockers (ICBs), and antibody-drug conjugates (ADCs), the treatment paradigm in both early stage and advanced TNBC has started to change [5]. Among all, ICBs have demonstrated promising results in terms of improving the rate and duration of the response and lowering the risk of disease progression in TNBC patients, especially when combined with chemotherapy [6,7,8]. Apart from application in metastatic TNBC patients, the combination of ICB and chemotherapy is starting to move to an early treatment setting for TNBC patients [9]. However, one major challenge associated with ICBs, especially when used in monotherapy, is the small fraction of patients deriving long-lasting benefits from the treatment, for the development of early or secondary resistance with ICBs is a relatively common event [5]. In patients, after achieving objective responses with ICBs, populations of tumor cells manage to escape from the immune response, reinforcing a microenvironment that enhances tumor progression and suppresses anti-tumor immunity [10]. Hence, a major effort is to hamper such resistance by means of modulating the tumor microenvironment (TME) to combat immune escape mechanisms. With the evidence of TNBC being one of the most immunogenic subtypes of breast cancer [4], one such approach would be to evoke active immune responses in the TME. These responses, especially when cancer-cell specific, would not only contribute to the elimination of cancer cells, but also act as an adjuvant strategy to passive immunotherapies via delaying the anticipated resistance mechanisms. Of the active immunotherapeutic agents, vaccines induce prolonged, sustainable, and, most importantly, specific responses to a targeted antigen (Ag) [10].

Although vaccines are widely used against infectious diseases, their efficacy in cancer prevention has been investigated in limited groups of infectious-based cancers, such as human papillomavirus (HPV) infection [11,12]. Thereafter, sipuleucel-T has been the first and the only cancer vaccine approved by the FDA for the treatment of cancer since 2010. Generally speaking, cancer vaccines may be effective both in a preventive setting by activating memory cells that prevent cancer recurrence, and in a therapeutic setting by amplifying the antitumor defense through enhancing T-cell responses to tumor-derived Ags [11,13]. The Ags should be selected so that they cover three main criteria: cancer cell-specific, highly immunogenic, and crucial for the survival of tumor cells; and able to be delivered through a number of platforms, including as peptides mixed with adjuvants, as Ag-presenting cells (APCs), or as nucleic acids [14]. Upon Ag presentation through MHC molecules on the surface of APCs, these cells migrate to secondary lymphoid organs, where they prime naïve T-cells and activate B-cells. The infiltration of the consequently mature T-cells into the TME as well as the presence of specific neutralizing antibodies in the circulation cause the effective destruction of the residual cancer cells. Tumor cell lysis releases cancer Ags in the TME, further reinforcing this immunity cycle and causing a persistent and sustained anti-tumor response [15]. It is worth mentioning that, since cellular immunity is central to the eradication of malignant cells, a major effort in the development of cancer vaccines is to specifically boost their CD8-activating potential through MHC class I epitopes.

Various types of vaccines with different Ag targets, delivery platforms, and administration routes have been investigated in solid tumors. While demonstrating minimal toxicity and inducing specific immune responses, the clinical benefit associated with the majority of these vaccines has been dismal. Several reasons can contribute to such an observation, including the development of further immune escape mechanisms through the down-regulation of TAAs or HLA molecules, resulting in suboptimal Ag presentation or T-cell exhaustion [16,17]. Moreover, an increase in the population of suppressive cells in the TME, including regulatory T-cells (Tregs) or myeloid-derived suppressor cells (MDSCs), has been associated with a poor response to immunotherapy [18,19]. Thus, it is important to optimize the process of vaccine development, including the choice of Ags and delivery platforms, as well as different combination therapy strategies to improve the outcomes of vaccine therapy.

Although preclinical studies have demonstrated promising results of cancer vaccines in TNBC, their clinical translation, safety, and efficacy have yet to be determined [11,20,21]. Thus, we aimed to systematically review the literature to provide a comprehensive update on the efficacy and safety of various vaccines against TNBC in the clinical setting.

## 2. Methods

The present systematic review was performed based on the Preferred Reporting Items for Systematic Reviews and Meta-Analyses (PRISMA) guidelines and the protocol was registered in the International prospective register of systematic reviews (PROSPERO) database (CRD42020206870).

### 2.1. Search Strategy, Eligibility Criteria, and Results

On 21 February 2021, a comprehensive search using PubMed, Scopus, Web of Science, Embase, Cochrane CENTRAL, and Google Scholar databases was performed, using a combination of the following keywords: triple negative breast cancer, TNBC, vaccine, and active immunization. The complete search strategy is available in supplementary documents. Results were restricted to English articles only, without any limitation on publication date. Additional results were also obtained through manual search. A further search update in the aforementioned databases was also performed on 5 July 2022 to obtain the most recent results.

Eligibility criteria included all clinical studies, including randomized and non-randomized clinical trials, cohorts, case studies, and case series, which evaluated a tumor vaccine in triple-negative breast cancer patients. Non-peer reviewed papers, or those not reporting a clinical endpoint for survival, progression of the disease, or safety of the vaccine were excluded. Also, studies of cancer vaccines performed on breast cancer patients which did not, qualitatively or quantitatively, report the efficacy and/or safety in triple negative breast cancer patient subset were excluded. Initially, title/abstract screening was done by at least two independent reviewers. After removing duplicated, irrelevant, and non-eligible results, full-text screening was conducted by at least two independent reviewers. In each stage any possible discrepancies were resolved through discussion or consensus with a third expert reviewer.

**Figure 1:**
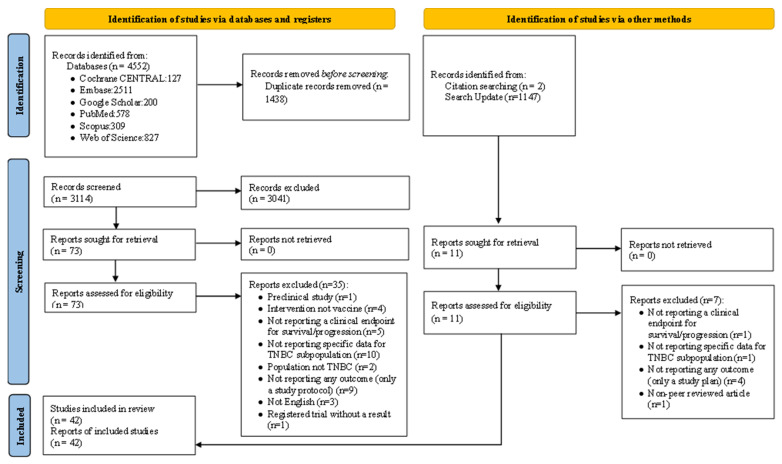
PRISMA flowchart of the study process.

In total, 4552 results were obtained through searching PubMed, Scopus, Web of Science, Embase, Cochrane CENTRAL, and Google Scholar databases using the pre-defined search strategy. After removing 1438 duplicate results, 3114 articles were screened in the initial screening step, of which 73 results were selected for full-text screening. During manual search (two studies) and search updating (two studies), four additional studies were included. Finally, 42 results were eligible to be included in this study. We also searched the clinicaltrials.gov database for relevant trials, yielding a total of 20 results that were not associated with any published articles. A summary of screening and study selection procedures, as well as reasons for exclusion, is depicted in Figure 1.

Of the 42 eligible studies, the following data were extracted through a predefined Google form: author name, publication year, study type and design, duration of the intervention, trial ID and phase, sample size per arm, tumor stage, vaccine type, formulation, dosage and route of administration, and clinical outcome measures of efficacy and safety. Where clinical outcomes were not directly expressed as numbers, we extracted the relevant data from graphs using WebPlotDigitizer online tool [22].

### 2.2. Characteristics of Included Studies

The total 42 included studies comprised 14 full-text articles, of which 6 results were randomized controlled trials (RCTs), 5 were non-randomized trials, and 3 were case reports. The remainder of results belonged to conference abstracts (28 results). Summary of study characteristics as well as detailed description of included studies are given in Table 1 and Table 2, respectively. Table 3 describes characteristics of eligible trials which do not currently have any published results.

### 2.3. Quality Assessment of Results

Two independent reviewers assessed the quality of included studies using the modified Jadad scale [23] or the JBI Critical Appraisal Tool for quasi-experimental studies [24] for reporting randomized and non-randomized clinical studies, respectively. Discrepancies were resolved by a third expert reviewer.

The quality score of the included randomized trials assessed by a Modified Jadad Scale ranged from 4 to 8, with a mean score and standard deviation (SD) of 6.5 ± 1.52. Three (60%) of the included studies were single-blind, only one (20%) study was double-blind, and one (20%) trial did not use blinding at all. Most of the included studies (66.67%) used appropriate blinding and randomization methods and described participants’ withdrawals. All studies described as randomized clearly stated inclusion/exclusion criteria, and mentioned the methods of assessing adverse effects and methods of analyzing data (Table S1, Supplementary files).

For non-randomized studies, the percent of “Yes” answers in each study ranged from 55.56% to 100%. All included studies stated “cause” and “effect,” pre- and post-intervention outcome measurement, participants’ follow-up adequacy, reliable outcome measurement method, and used the same way to measure outcomes in any comparisons. A majority of studies (60%) described statistical methods. Less than half (40%) of the non-randomized studies used similar participants in all comparisons, and only 20% of studies had a control group and exposed participants to similar treatments (Table S2, Supplementary files).

**Table 1:** Cancer vaccines currently evaluated in TNBC for safety/efficacy, based on included results. Non-randomized trials are marked with asterisks.

Vaccine Name	Trial ID	Phase	Platform	Target Antigen	Adjuvant	Co-Therapies	Population	Outcomes	Reference
AE37	NCT00524277	2	Peptide	HER2 ^1^	GM-CSF ^2^	-	Node positive, high-risk node-negative BC ^3^	DFS ^4^, safety	Full-text [25,26] Abstract [27,28,29,30,31]
GP2	NCT00524277	2	Peptide	HER2	GM-CSF	-	Node positive, high-risk node-negative BC	DFS, safety	Full-text [26] Abstract [32]
Nelipepimut-S	NCT01570036	2	Peptide	HER2	GM-CSF	Trastuzumab	Invasive BC, HER2-low	DFS, safety, IR ^5^	Full-text [33,34] Abstract [35,36,37,38]
Tecemotide	EudraCT2011-004822-85	2	Peptide	MUC1 ^6^	Monophosphoryl lipid A	Chemotherapy (E ^7^ C ^8^ + D ^9^)	Early invasive BC	RCB ^10^, pCR ^11^, safety, QoL ^12^	Full-text [39]
AS/OBI-821	NCT01516307	2	Peptide	Globo-H	OBI-821	Cyclophosphamide	m ^13^ BC	PFS ^14^, OS ^15^, IR, safety	Full-text [40]
PVX-410	NCT02826434 *	1	Peptide	XBP1 ^16^, CD138, CS-1	Poly-ICLC, montanide	Durvalumab	e ^17^ TNBC	Safety, IR	Abstract [41]
	NCT03362060 *	1	Peptide	XBP1, CD138, CS-1	Poly-ICLC, montanide	Pembrolizumab	mTNBC	Safety, IR, CBR ^18^, DCR ^19^, DoR ^20^, PFS, OS	Abstract [42]
H/K-HELP	UMIN000003489 *	1	Peptide	Survivn	OK-432, montanide	-	Advanced solid tumors, including mTNBC	IR, safety	Full-text [43] Abstract [44]
Personalized peptide vaccination (PPV)	UMIN000001844 *	2	Peptide	Up to 4 antigens	Montanide ISA 51	SoC ^21^ chemotherapy/RT ^22^	mBC	IR, safety, PFS, OS	Full-text [45] Abstract [46,47,48]
KRM-19	UMIN000014616 *	2	Peptide	Combination 19 antigens	Montanide ISA 51	-	mTNBC	Safety, PFS, IR	Full-text [49] Abstract [50]
Multipeptide active immunotherapy	N/S *	1	Peptide	Combination 22 antigens	N/S	Chemotherapy (Oxaliplatin, doxorubicin)	Ovarian cancer, soft sarcoma, pancreatic cancer, TNBC	Safety, IR	Abstract [51]
Tumor lysate-pulsed DC ^23^ vaccine	NCT01431196 * EudraCT 2009-017402-36	2	Dendritic Cell	Whole tumor cells	-	NAC ^24^ (dd ^25^ EC+ D)	eBC, HER2-negative	Safety, pCR, OS, IR	Full-text [52] Abstract [53,54]
Antigen-loaded DC vaccine	NCT02018458	1	Dendritic Cell	Cyclin B1, WT1 ^26^, CEF	-	dd A ^27^ C + T ^28^ Cb ^29^	Locally advanced TNBC	Safety, local pCR, IR	Abstract [55,56,57]
RO7198457 (iNEST)	NCT03289962 *	1	RNA	Personalized neoantigens	-	Atezolizumab	Locally advanced/m solid tumors	Safety, IR, ORR ^30^	Abstract [58]
p53MVA	NCT02432963 *	1	DNA (MVA ^31^)	p53	-	Pembrolizumab	Advanced breast, pancreatic, hepatocellular, H&N ^32^ tumors	Safety, IR, RR ^33^	Full-text [59,60] Abstract [61]
NANT cancer vaccine (NCV)	NCT03387085 *	1	DNA (Adenovirus/yeast)	CEA ^34^, MUC1, brachyury, HER2, and RAS	-	Low dose chemo, SBRT ^35^, N-803, avelumab, haNK cells ^36^	mTNBC	Safety, ORR, DCR ^37^, PFS, OS	Abstract [62,63,64]
Elenagen	trial #506, Protocol E001 *	1/2	DNA (Plasmid)	p62	-	SoC chemo (C/M ^38^/F ^39^)	Metastatic breast, colon, renal, lung, and ovarian cancers, and melanoma	Safety, RR	Full-text [65,66]

^1^ HER2: human epidermal growth factor receptor 2; ^2^ GM-CSF: granulocyte-macrophage colony-stimulating factor; ^3^ BC: breast cancer; ^4^ DFS: disease-free survival; ^5^ IR: immune response; ^6^ MUC1: mucin 1; ^7^ E: epirubicin; ^8^ C: cyclophosphamide; ^9^ D: docetaxel; ^10^ RCB: residual cancer burden; ^11^ pCR: pathological complete response; ^12^ QoL: quality of life; ^13^ m: metastatic; ^14^ PFS: progression-free survival; ^15^ OS: overall survival; ^16^ XBP1: X-box binding protein 1; ^17^ e: early; ^18^ CBR: clinical benefit rate; ^19^ DCR: disease control rate; ^20^ DoR: duration of response; ^21^ SoC: standard of care; ^22^ RT: radiotherapy; ^23^ DC: dendritic cell; ^24^ NAC: neoadjuvant chemotherapy; ^25^ dd: dose-dense; ^26^ WT1: Wilm’s tumor 1; ^27^ A: doxorubicin; ^28^ T: paclitaxel, ^29^ Cb: carboplatin; ^30^ ORR: objective response rate; ^31^ MVA: modified vaccinia virus Ankara; ^32^ H&N: head and neck; ^33^ RR: response rate; ^34^ CEA: carcinoembryonic antigen; ^35^ SBRT: stereotactic body radiation therapy; ^36^ haNK cells: high-affinity natural killer cells; ^37^ DCR: disease control rate; ^38^ M: methotrexate; ^39^ F: fluorouracil.

**Table 2:** Summary of study details of included studies. Full-text articles are marked with asterisks. All data, except safety, are presented for TNBC cohort of each trial.

Vaccine Name	Study	# Patients (VG ^1^:CG ^2^)	Dosing, Route, Intervals	Follow-up Duration (m ^3^/w ^4^/d ^5^)	Key Observations (Efficacy)	Key Observations (Safety)
AE37	Brown et al., 2020 *	45 (21:24)	AE37 (500 µg) + GM-CSF (125 µg), x6 q3-4w (VG)/ GM-CSF (125 µg) x6 q3-4w (CG) *ID* ^6^ 4 boosters q6m	59.9 m	No statistically significant difference in 5-y ^7^ DFS ^8^ (*p* = 0.226, HR ^9^ = 0.443); signs of DFS improvement for advanced-stage TNBC patients (*p* = 0.078, HR = 0.184)	Safe and well-tolerated, majority of AE ^10^s of grade 1, no >grade 3 toxicities
	Peace et al., 2017	42 (21:21)		55 m	Statistically significant difference in DFS (*p* = 0.0478, HR = 0.26)	Safe and well-tolerated, no >grade 3 AEs
	Peace et al., 2017	N/S		46.7 m	Signs of DFS improvement for advanced-stage TNBC patients (*p* = 0.054)	N/S ^11^
	Mittendorf et al., 2016 *	50 (25:25)		25 m	No statistically significant difference in 5-y DFS (*p* = 0.12, HR = 0.403)	Maximum local AEs grade 1/2, no >grade 3 systemic AEs
	Greene et al., 2015	51 (25:26)		N/S	No statistically significant difference in 5-y DFS (*p* = 0.65, HR = 0.42)	N/S
	Mittendorf et al., 2014	50 (25:25)		N/S	No statistically significant difference in 5-y DFS (*p* = 0.12, HR = 0.40)	Safe and well-tolerated, no grade 3 local AEs, 1 pt ^12^ had grade 3 systemic AE
	Mittendorf et al., 2012	36 (13:23)		22.3 m	No statistically significant difference in 5-y DFS (*p* = 0.23)	N/S
GP2	Brown et al., 2020 *	N/S	GP2 (500 µg) + GM-CSF (125 µg), x6 q3-4w (VG)/ GM-CSF (125 µg), x6 q3-4w (CG) *ID* 4 boosters q6m	41.7 m	No statistically significant difference in RR ^13^ (HR~1.42)	Safe and well-tolerated, majority of AESs of grade 1, no >grade 3 AEs
	Trappey et al., 2013	N/S		24 m	No statistically significant difference in RR (*p* = 0.251)	Maximum local and systemic AEs similar between groups, no grade 3 systemic AEs
Nelipepimut-S	Chick et al., 2021 *	99 (55:44)	trastuzumab (LD ^14^ 8mg/kg, MD ^15^ 6 mg/kg) q3w for 1 y + NPS (1000 µg) + GM-CSF (250 µg), x6 q3w, starting with 3^rd^ trastuzumab dose (VG)/ trastuzumab (LD 8mg/kg, MD 6 mg/kg) q3w for 1 y + GM-CSF (250 µg), x6 q3w, starting with 3^rd^ trastuzumab dose (CG) *ID* 4 boosters q6m	36 m	Statistically significant differences in 36-m DFS of total pts (*p* = 0.01, HR = 0.25), HER2 IHC ^16^ 1+ pts (*p* = 0.01, HR = 0.17), HLA-A24+ pts (*p* < 0.01, HR = 0.08), pts with prior NAC ^17^ (*p* < 0.01, HR = 0.21)	N/S
	Clifton et al., 2020 *	97 (53:44)		26.1 m	Statistically significant difference in 24-m DFS (*p* = 0.01, HR = 0.25)	Safe and well-tolerated, no added cardiac or overall AEs, no grade 4/5 AEs
	Clifton et al., 2019	97 (53:44)		N/S	Statistically significant difference in 36-m DFS of pts (*p* = 0.013, HR = 0.262), pts with prior NAC (*p* = 0.013, HR= 0.226), HER2 IHC 1+ pts (*p*= 0.014, HR= 0.178), ≥51 y-old pts (*p*= 0.004, HR= 0.144), stage I/II pts (*p* = 0.006)	N/S
	Hickerson et al., 2019	N/S		25.7 m	Statistically significant difference in 24-m DFS (*p* = 0.013, HR = 0.26)	Safe, no added cardiac or overall AEs, no grade 4/5 AEs
	Hickerson et al., 2019	N/S		19.4 m	Statistically significant difference in 24-m DFS (*p* = 0.02, HR = 0.26)	Safe, no grade 4/5 AEs
	Hale et al., 2018	N/S		18.8 m	Statistically significant difference in 24-m DFS in total (HR = 0.29) and HLA-A24+ pts (*p* = 0.02, HR = 0.08)	N/S
Tecemotide	Singer et al., 2020 *	116 (62:54)	E ^18^ C ^19^ (90 mg/m^2^ and 600 mg/m^2^ q3w, respectively), and D ^20^ (100 mg/m^2^, 3qw) or vice-versa, x4 + cyclophosphamide (300 mg/m^2^) 3 days before vaccine + tecemotide (930 µg), x8 qw (VG)/ EC (90 mg/m^2^ and 600 mg/m^2^ q3w, respectively), and D (100 mg/m^2^, 3qw) or vice-versa, x4 (CG) *SC* ^21^ 1 booster 2-3 weeks after last cycle	29.5 w	No statistically significant differences in either RCB ^22^ or pCR ^23^ (data not disclosed)	No different AEs between CG and VC, except for injection site AEs
AS/OBI-821	Huang et al., 2020 *	45 (28:17)	cyclophosphamide (300 mg/m^2^) 3 days before vaccine at weeks 1, 5, 9, 13, 17, 25, 37 + AS/OBI-821 (30 µg/100 µg) at weeks 1, 2, 3, 5, 9, 13, 17, 25, 37 (VG)/ cyclophosphamide (300 mg/m^2^) 3 days before vaccine at weeks 1, 5, 9, 13, 17, 25, 37 + placebo at weeks 1, 2, 3, 5, 9, 13, 17, 25, 37 (CG) *SC*	22.3 m	No statistically significant differences in PFS ^24^	All injection-site and most non-injection site AEs of grade 1/2, 2 hypersensitivity and 1 fever cases in VG
PVX-410	Isakoff et al., 2022	19 (nr ^25^)	pembrolizumab 200 mg, q3w starting at 2^nd^ vaccine dose+ PVX-410 (800 µg), x5 qw 2 boosters at w10 and 28 *SC*	36.8 m	PFS = 2.3 m, OS ^26^ = 2.3 m; best overall response was SD ^27^ in 47% of pts, CBR ^28^ = 31.6%, no pts had CR ^29^ /PR ^30^	Mostly grade 2 AEs, 2 grade 3 and 1 grade 4 AEs were attributable to pembrolizumab, no grade 5 AEs
	Isakoff et al., 2020	22 (nr)	durvalumab 1500 mg, x2, with 4^th^ and 6^th^ vaccine doses + PVX-410 (800 µg), x6 q2w *SC*	15.4 m	PVX-specific responses in 10/12 pts up to w 14 and persisted up to 6 m; 1/22 pt had local and 3/22 pts had metastatic recurrence, 2/22 pts died	No DLT ^31^ s, mostly injection site AEs, 2 grade 3 diarrhea and hyponatremia, respectively, no grade 4/5 AEs
H/K-HELP	Nishimura et al., 2011	N/S	H/K-HELP x4 q2w *SC*	N/S	Complete regression of resistant cervical node recurrence in TNBC pt	N/S
	Ohtake et al., 2014 *	1		71 d		
Personalized peptide vaccination (PPV)	Toh et al., 2012	8 (nr)	1^st^ cycle: PPV (3mg/each peptide), x6 qw; 2^nd^ cycle: PPV (with re-selected peptides, 3 mg/each peptide), x6 q2w 3^rd^ cycle: PPV (with re-selected peptides, 3 mg/each peptide), q4-8w continued until disease progression *SC*	N/S	Total population: 2 pts had CR and 1 pt had PR; no significant difference between TNBC and other subtypes in clinical responses	No vaccine-related SAE ^32^ s
	Takahashi et al., 2012	8 (nr)		N/S	6/8 pts received ≥6 vaccine doses; 5/6 pts reached SD, 1/6 pt reached CR	N/S
	Toh et al., 2013	14 (nr)		20.7 m	Median PFS and OS of TNBC were 8.3 m and 12 m, respectively	No vaccine-related SAEs
	Takahashi et al., 2014 *	18 (nr)		N/S	1/18 pt had CR, 1/18 pt had PR; median PFS 7.5 m, median OS 11.1 m; no significant PFS and OS difference between PPV mono (16 pts)/combination chemotherapy (2 pts) (*p* = 0.467 and 0.347, respectively)	No vaccine-related SAEs
KRM-19	Toh et al., 2020 *	14 (nr)	KRM-19 (19 mg/mL, 1 mg of each peptide), x6 qw *SC*	12 m	6/14 pts had SD, 8/14 had PD ^33^; median PFS: 1.5 m in 14/14 pts, and 5.8 m in 10/14 pts completing vaccination series; median OS: 11.5 m in 14/14 pts, and 24.4 m in 10/14 pts completing vaccination	Most commonly injection site AEs (9 pts) and lymphocytopenia (4 pts), 5 pts with grade 3, 0 with grade 4, and 2 with grade 5 AEs
	Toh et al., 2017	10 (nr)		N/S	1/8 pt had PR, 4/8 pts had SD, 3/8 pts had PD; 12.5% ORR ^34^, 62.5% CBR	10/10 pts had grade 1/2 injection site AEs, 5/10 pts had grade 2/3 liver function disorder, 3/10 pts had grade 2 bone marrow suppression, 1/10 pt had grade 2 nausea
Multipeptide active immunotherapy	Marquez-Manriquez et al., 2018	10 (nr)	oxaliplatin + doxorubicin (low-dose), x4 q2w + 22 peptides, x4 qw *SC*	N/S	All pts entered remission according to CT ^35^, all pts had significant CD8 response against all peptides	Well-tolerated in all pts, minimal injection site AEs
Tumor lysate-pulsed DC ^36^ vaccine	Santisteban et al., 2021 *	30 (nr) (17:13)	NAC (dd ^37^ EC x4 → D x4) + DCV ^38^, x5 q3w+ x1 the day after surgery + RT ^39^+ DCV, x4 q2m (VG)/NAC (ddEC x4 → D x4) (CG) *ID*	8 y	TNBC pts: pCR 50% (VG) vs. 30.7% (CG); *p* = 0.255	No difference in grade 3/4 NAC AEs between groups, no grade ≥ 3 vaccine-relate AEs
	Elarre et al., 2016	30 (nr)		N/S	TNBC pts: pCR 67% (VG) vs. 17% (CG); increased TIL ^40^s observed in TNBC pts unlike other subtypes (*p* = 0.01)	N/S
	Urrizola et al., 2020	N/S		7.52 y	Total pts (83): statistically significant pCR improvement (p = 0.03) TNBC pts: pCR 50.0% (VG) vs. 30.7% (CG)	No difference in grade 3/4 NAC AEs between groups, no grade ≥ 3 vaccine-relate AEs
Antigen-loaded DC vaccine	O’Shaughnessy et al., 2020	10 (nr)	ddA ^41^ C + T ^42^ Cb ^43^ + DCV (x4 pre-surgery, x3 post-surgery) *IT* ^44^, *SC*	N/S	4/10 pts achieved pCR, 3/10 pts had local macroscopic residual disease, 3/10 pts had MRD ^45^	N/S
	Palucka et al., 2018	10 (nr)		12	All pts received 4 pre-surgery doses, 7/10 pts received all vaccine doses; 4/10 pts achieved pCR, 3/10 pts had local macroscopic residual disease, 3/10 pts had MRD	N/S
	O’Shaughnessy et al., 2016	10 (nr)		N/S	All pts received 4 pre-surgery doses, 4/10 pts received all vaccine doses; 5/10 pts achieved pCR, 3/10 pts had local macroscopic residual disease, 2/10 pts had MRD	9/10 pts had grade 1/2 injection site AEs
RO7198457 (iNEST)	Lopez et al., 2020	24 (nr)	atezolizumab (1200 mg) q3w+ RO7198457, x9 q1/2w for 12w (induction stage) and q24w (maintenance stage)	N/S	ORR = 4% in the TNBC cohort, and 8% in total pt population (108)	Majority of AEs of grade 1/2, no DLTs
p53MVA	Chung et al., 2019 *	7 (nr)	p53MVA (5.6*10^8^ pfu ^46^), x3 q3w + pembrolizumab (200 mg), x7 q3w *IM* ^47^	N/S	1/7 pt failing all prior therapy lines had regression of cutaneous metastases (pCR), 5/7 pts were removed from the study due to PD at week 10, 1/7 pt had SD for 30 w	1/11 pts grade 3 adrenal insufficiency, 2/11 pts LFT ^48^ rise, 1/11 pts grade 5 myocarditis (all mainly related to pembrolizumab)
	Chung et al., 2018	N/S		N/S	1 pt had durable p53-specific CD8 responses along with pCR and SD for >6 m, 1 pt was on study for 35 w, 2 pts had rapidly PD	N/S
	Yuan et al., 2017 *	1 (case report)		33 w	Sustained (6 m) and complete regression of cutaneous metastases after 9 w of treatment, minimal dermal relapse at week 33	Grade 2 nausea, grade 1 vomiting, grade 1 skin rash (possibly related to pembrolizumab)
NANT cancer vaccine (NCV)	Nangia et al., 2019	9 (nr)	Low-dose metronomic chemoradiation + N-803 +PD-L1 ^49^ inhibitor + haNK cells ^50^ + NCV (3w cycle) *N/S route of administration*	N/S	DCR ^51^ = 78% (7/9 pts with CR + PR + SD); ORR = 56% (5/9 pts with PR + CR); CR in 2 pts (22%)	4/9 pts had 8 grade ≥3 AEs (of which 2 pts had haNK-associated SAEs)
	Nangia et al., 2019	8 (nr)		N/S	1/8 pt had CR, 2/8 pts had PR	all pts had at least 1 grade ≥3 AEs, 2/8 pts had grade ≥ 3 haNK-associated AEs
	Carlson et al., 2018	3 (nr)		N/S	2/3 pts had PR	4 hematologic DLT’s were observed and managed with dose reduction of cisplatin
Elenagen	Ponomarenko et al., 2020 *	1 (case report)	Elenagen (1 mg), x5 qw, then q3w until disease progression ± CM ^52^ F ^53^ (C 600 mg/m^2^, M 40 mg/m^2^, F 600 mg/m^2^), days 1st and 8th q2w *IM*	N/S	PFS = 19 w, 33% partial tumor regression	Grade ½ nausea, grade 2/3 leukopenia and neutropenia
	Ponomarenko et al., 2017 *	4 (nr)		N/S	In monotherapy: 2/4 pts had PD; 2/4 pts had SD for 8 & 32 w; in Elenagen + CMF: prolongation of SD in pts with PD (for 24 w) or those with SD (24 w)	Safe with no DLTs/SAEs, only grade 1 injection site AEs, nausea, fatigue, fever

^1^ VG: vaccine group; ^2^ CG: control group; ^3^ m: month; ^4^ w: week; ^5^ d: day; ^6^ ID: intradermal; ^7^ y: year; ^8^ DFS: disease-free survival; ^9^ HR: hazard ratio; ^10^ AE: adverse event; ^11^ N/S: not specified; ^12^ pt: patient; ^13^ RR: recurrence risk; ^14^ LD: loading dose; ^15^ MD: maintenance dose; ^16^ IHC: immunohistochemistry; ^17^ NAC: neoadjuvant chemotherapy; ^18^ E: epirubicin; ^19^ C: cyclophosphamide; ^20^ D: docetaxel; ^21^ SC: subcutaneous; ^22^ RCB: residual cancer burden; ^23^ pCR: pathological complete response; ^24^ PFS: progression-free survival; ^25^ nr: non-randomized; ^26^ OS: overall survival; ^27^ SD: stable disease; ^28^ CBR: clinical benefit rate; ^29^ CR: complete response; ^30^ PR: partial response; ^31^ DLT: dose-limiting toxicity; ^32^ SAE: severe adverse event; ^33^ PD: progressive disease; ^34^ ORR: objective response rate; ^35^ CT: computed tomography; ^36^ DC: dendritic cell; ^37^ dd: dose-dense; ^38^ DCV: dendritic cell vaccination; ^39^ RT: radiotherapy; ^40^ TIL: tumor infiltrating lymphocyte; ^41^ A: doxorubicin; ^42^ T: paclitaxel; ^43^ Cb: carboplatin; ^44^ IT: intratumoral; ^45^ MRD: microscopic residual disease; ^46^ pfu: plaque-forming units; ^47^ IM: intramuscular; ^48^ LFT: liver function test; ^49^ PD-L1: programmed death-ligand 1; ^50^ ha-NK cells: high-affinity natural killer cells; ^51^ DCR: disease control rate; ^52^ M: methotrexate; ^53^ F: fluorouracil.

**Table 3:** Cancer vaccines in TNBC, based on unpublished data (registered trials). Non-randomized trials are marked with asterisks.

Vaccine Name	Trial ID	Phase	Platform	Target Antigen	Adjuvant	Co-Therapies	Disease Stage	Primary Outcome(s)	Status
AE37	NCT04024800	2	Peptide	HER2 ^1^	GM-CSF ^2^	Pembrolizumab	IV	Recommended dose, ORR ^3^	Active, not recruiting
Galinpepimut-S	NCT03761914	1/2	Peptide	WT1 ^4^	N/S ^5^	Pembrolizumab	IV	Safety, ORR, CRR ^6^	Active, not recruiting
MUC1 ^7^ vaccine	NCT00986609	1	Peptide	MUC1 ^7^	Poly-ICLC	-	I-III	IR ^8^	Completed
CDX-1401	NCT02661100	1/2	Peptide	NY-ESO-1 ^9^	Poly-ICLC	Pembrolizumab	Advanced TNBC	DLT ^10^	Withdrawn
P10s-PADRE	NCT02938442	1/2	Peptide	GD2/LeY ^11^	Montanide ISA 51	NAC ^12^ (A ^13^ C ^14^ T ^15^)	I-III	Safety, pCR ^16^	Recruiting
NGcGM3/VSSP	RPCEC00000218	1/2	Peptide	NGcGM3	VSSP ^17^	Nimotuzumab	IV	OS ^18^	Pending recruitment
FR-α ^19^ vaccine	NCT02593227	2	Peptide	FR-α	GM-CSF	Cyclophosphamide	I-III	IR	Completed
	NCT03012100	2	Peptide	FR-α	GM-CSF	Cyclophosphamide	I-III	DFS ^20^	Recruiting
HER2/MUC1 vaccine	NCT00640861	1	Peptide	HER2/MUC1	CpG oligodeoxynucleotide, incomplete Freund’s adjuvant, sargramostim	-	II-III	IR, safety	Completed
Neoantigen long peptide vaccine	NCT02427581	1	Peptide	Personalized neoantigens	Poly ICLC	-	e ^21^ TNBC	Safety, IR	Withdrawn
Apoptotic tumor cell-pulsed DC ^22^ vaccine	ChiCTR-IPR-15005955	1	Dendritic Cell	Whole tumor cells	-	-	II-IV	IR, tumor markers, PFS ^23^	Pending recruitment
HER2/HER3 ^24^ loaded DC vaccine	NCT04348747	2	Dendritic Cell	HER2, HER3	-	Pembrolizumab	IV (brain metastases)	CNS ^25^ ORR	Recruiting
Neoantigen-pulsed DC vaccine	NCT04105582	1	Dendritic Cell	Personalized neoantigens	-	-	N/S	Safety	Completed
	NCT04879888								
NANT cancer vaccine (NCV)	NCT03175666 *	1/2	DNA (Adenovirus/yeast)	CEA ^26^, MUC1, brachyury, and RAS	-	Avelumab, bevacizumab, N-803, chemotherapy, SBRT ^27^, haNK cells ^28^	IV	Safety, ORR	Withdrawn
	NCT03554109	2	DNA (Adenovirus/yeast)	CEA, MUC1, brachyury, and RAS		Avelumab, N-803, chemotherapy, haNK cells	II/III	pCR	Withdrawn
PF-06936308	NCT03674827	1	DNA (Adenovirus)	3 TAA ^29^s (non-disclosed)	-	-	IV	CBR ^30^, safety	Terminated
Polyepitope Neoantigen DNA Vaccine	NCT02348320	1	DNA (Plasmid)	Personalized neoantigens	-	-	I-III	Safety	Completed
	NCT03199040	1	DNA (Plasmid)	Personalized neoantigens	-	Durvalumab	I-III	Safety	Active, not recruiting
STEMVAC	NCT05455658 *	2	DNA (Plasmid)	CD105, Yb-1 ^31^, SOX2, CDH3, MDM2 ^32^	-	GM-CSF	I-III	IR	Not yet recruiting

^1^ HER2: human epidermal growth factor receptor 2; ^2^ GM-CSF: granulocyte-macrophage colony-stimulating factor; ^3^ ORR: objective response rate; ^4^ WT1: Wilm’s tumor 1; ^5^ N/S: not specified; ^6^ CRR: complete response rate; ^7^ MUC1: mucin-1; ^8^ IR: immune response; ^9^ NY-ESO-1: New York esophageal squamous cell carcinoma-1; ^10^ DLT: dose-limiting toxicity; ^11^ LeY: Lewis Y; ^12^ NAC: neoadjuvant chemotherapy; ^13^ A: doxorubicin; ^14^ C: cyclophosphamide; ^15^ T: paclitaxel; ^16^ pCR: pathological complete response; ^17^ VSSP: Very small size proteo-liposome; ^18^ OS: Overall survival; ^19^ FRα: Folate Receptor-α; ^20^ DFS: Disease-free survival; ^21^ e: early; ^22^ DC: dendritic cell; ^23^ PFS: progression-free survival; ^24^ HER3: human epidermal growth factor receptor 3; ^25^ CNS: central nervous system; ^26^ CEA: carcinoembryonic antigen; ^27^ SBRT: stereotactic body radiation therapy; ^28^ haNK cells: high-affinity natural killer cells; ^29^ TAA: tumor-associated antigen; ^30^ CBR: clinical benefit rate; ^31^ Yb-1: Y-box binding protein 1; ^32^ MDM2: Murine double minute 2.

## 3. Results

The vaccines evaluated in TNBC patients can be divided into the three major subtypes of peptide-based, cell-based, and nucleic acid (RNA/DNA)-based. As is the case for vaccines under development for the majority of cancer types, peptide vaccines targeting a tumor-associated Ag (TAA) comprise the largest share of formulations (59%); however, DNA and RNA vaccines targeting multiple TAAs or a personalized set of neoantigens (neoAgs) are gaining increasing popularity (26% of all formulations). While most of the vaccines are currently in phase 1 or 2 studies, very few vaccines (only 2%) with promising results have recently jumped to randomized phase 3 trials. Besides the safety of the formulation, some studies have assessed preliminary clinical outcomes such as induction of Ag-specific immune responses in early phase I studies. In terms of safety, almost all formulations under development have been associated with favorable safety profiles and tolerable set of adverse events (AEs) in their associated follow-up time courses (Table 2). While immune response profiles of patients following vaccine administration have been promising in most of the preliminary reports, the clinical benefit of many vaccines is still under investigation in the TNBC population. However, a large number of the vaccines already investigated in this population have shown promising results in terms of their clinical benefit. Due to the heterogeneity of clinical outcomes reported and study designs, we were unable to conduct a meta-analysis; we thereby discuss different vaccines and the results of their associated clinical studies in detail in the following sections, based on vaccine types and targets. 

### 3.1. Peptide Vaccines

Peptide vaccines directly evoke immune responses against one or a set of tumor Ag(s) upon administration. While tumor-associated Ags (TAAs) are partially expressed in normal tissues as well as on tumor cells, tumor-specific Ags (TSAs) are exclusive to cancer tissues, and thus, vaccines targeting TSAs are considered to have more acceptable safety profiles. Moreover, the elimination of T-cells targeting TAAs is probable due to central tolerance mechanisms. Thus, the development of peptide vaccines has been moving toward “more specific, more personalized” or “neoAg-based” approaches in recent years, which have demonstrated more promising results than the classical TAA-targeting peptide vaccines. It should be noted that the addition of adjuvants or fusion to carrier proteins is crucial to the formulation of peptide vaccines, since many tumor Ags are associated with low immunogenicity [67]. Peptide vaccines are usually administered via intradermal or subcutaneous routes, and are sometimes combined with other treatment modalities such as chemo/radio/immunotherapies to enhance their efficacy.

#### 3.1.1. Shared Ag Vaccines

##### 3.1.1.1. HER2

While HER2/neu overexpression is observed in only a subset of breast tumors, this tyrosine-kinase receptor is normally expressed in a majority of breast and other epithelial carcinomas [68]. However, targeting HER2 via monoclonal antibodies, e.g., trastuzumab, has been a main therapeutic tool in HER2-overexpressing breast cancer, as proven by the NSABP B-47 trial, in which no survival benefit was observed in HER2-low expressing patients [69]. TNBC tumors demonstrate varying degrees of HER2 expression, from HER2 absence (IHC score of 0) to low expression (IHC score of 1+/2+) [70]. Several characteristics make HER2 an attractive candidate for active immunotherapy, including its well-defined nature and the many immunogenic epitopes it possesses. Therefore, HER2-based vaccines may be efficient at eliciting immune responses even in patients with low HER2 expression, including subsets of TNBC patients [71].

AE37

AE37 is a peptide, HER2-derived vaccine which is actually a hybrid of the MHC class II, intracellular HER2 epitope AE36 (amino acids 776–790) and the key 4- amino acid peptide of the Ii protein, namely the Ii-Key peptide (LRMK). LRMK boosts the binding properties of epitopes to MHC class II molecules, since class II epitopes have a lower binding affinity compared to their class I counterparts [72]. AE36 binds to MHC class II, thus triggering CD4+ helper T immune responses and mediating long-term cellular immunity with the further activation of CD8+ T-cells [73]. This key characteristic discriminates the AE37 vaccine from other HER2-based vaccines, which mainly stimulate CD8+ responses. Moreover, AE37 may be beneficial to patients regardless of its MHC status, unlike MHC class I-based vaccines which are only useful in patients with specific class I alleles. With preclinical evidence of AE37-mediated CTL responses in tumor models [74,75], this vaccine has entered phase I and II trials in breast and prostate cancers [76,77].

Proving to be safe and immunogenic in a phase I trial of breast cancer patients [76], a primary report published by Mittendorf et al. in 2012 on a phase II, randomized controlled trial of the AE37 vaccine in the adjuvant setting in disease-free patients with node-positive and high-risk node-negative breast cancer to prevent disease recurrence (NCT00524277) provided promising evidence of efficacy of the vaccine in HER2-low expressing breast cancer subtypes, including TNBC patients. With GM-CSF as a vaccine adjuvant, patients were randomized to receive either AE37+GM-CSF or GM-CSF alone through monthly intradermal inoculations for 6 months, followed by four semi-annual booster inoculations. While no statistically significant difference in disease recurrence was observed with the administration of the vaccine overall and in subgroup analyses, a 68% recurrence risk reduction was observed in the HER2-low expressing and TNBC subgroups, which proved a greater benefit as compared to the whole study population, who experienced a 49% risk reduction [27]. These early analyses suggested a potential benefit of AE37 vaccination, particularly in HER2-low expressing and TNBC subtypes. A further follow-up of this actively recruiting trial revealed the even greater efficiency of AE37 in TNBC patients compared to the HER2-low expressing subgroup (60% vs. 40% risk reduction), while still no statistically significant difference was observed. Further, this report provided the primary safety data of AE37, claiming it to be well-tolerated with only one patient experiencing grade 3 systemic toxicity and no grade 3 local toxicity [28]. In its final pre-specified analysis one year from the last patient enrolled, hormone-receptor negative as well as TNBC patients were demonstrated to have gained the most benefit from vaccination, with 33% and 35% reductions in recurrence risk, respectively. The vaccine was again well-tolerated, with mainly grade 1 local and systemic toxicities, and effectively induced HER2-specific immune responses, however, a further final report of the study’s primary analysis with a median follow-up duration of 25 months also reported no statistically significant reduction in recurrence risk upon vaccine inoculation [25]. Overall, the results, although non-significant, outlined the importance of target population selection for administering the AE37 vaccine, focusing on TNBC patients.

According to the fact that the efficacy of cancer vaccines may require more time to be demonstrated in breast cancer patients due to late-occurring recurrences, the extended follow-up data of this phase II trial collected after 55 months demonstrated statistically significant DFS improvement in TNBC patients and patients with a stage IIB/III disease and low HER2 expression [31]. However, a further report of the trial in which both AE37 and GP2 were administered to breast cancer patients failed to demonstrate a statistically significant difference in recurrence risk between the AE37 and control arms after a median follow-up of about 60 months (*p* = 0.226); advanced stage TNBC patients seemed to derive more benefit from AE37 vaccination than other subgroups (*p* = 0.078) [26,30].

Considering the immunostimulatory mode of action of AE37, which involves both CD4+ and CD8+ responses, this vaccine can potentially serve as an adjuvant immunotherapy along with other CD8+-stimulating vaccines or immune checkpoint inhibitors. Accordingly, a recent clinical trial on the concurrent use of AE37 (without GM-CSF, every 21 days for 5 doses) and pembrolizumab (every 21 days for 2 years) in metastatic TNBC patients is registered (NCT04024800), with the aim of establishing the recommended dose, safety, and efficacy of AE37 in combination with pembrolizumab, a PD-1 checkpoint inhibitor monoclonal antibody.

GP2

GP2 is a sub-dominant epitope of the HER2 transmembrane domain (amino acids 654–662), which has the advantage of preventing potential T-cell anergy compared to dominant epitopes. However, it is HLA-A2 restricted and only beneficial to HLA-A2-positive breast cancer patients. GP2 has proved to be safe and immunogenic in breast cancer patients in previous phase I studies [78,79]. In the randomized trial mentioned in the AE37 subsection (NCT00524277) on clinically disease-free breast cancer patients with node-positive or high-risk node-negative disease, GP2 was administered to HLA-A2-positive patients in combination with GM-CSF as a vaccine adjuvant in 6 monthly intradermal inoculations as the primary vaccine series (PVS), followed by booster inoculations every 6 months. The placebo arm received GM-CSF only. Adverse events were majorly limited to grade 1 toxicities with comparable rates to that of the placebo arm, implying that these adverse events were most likely due to GM-CSF administration [80]. In their interim-analysis report after 24 months of follow-up, a non-significant risk reduction was observed following GP2 administration in TNBC patients (*p* = 0.28) which was comparable to that of the whole study population (*p* = 0.41) or HER2-overexpressing patients (*p* = 0.25) [32]. However, in the final analysis after about 42 months of follow-up, HER2-overexpressing patients were the subset deriving the most benefit from GP2 (*p* = 0.052) compared to TNBC patients (HR~1.42 in the TNBC subset, derived using WebPlotDigitizer online tool) [26,30]. Of interest, these HER2-overexpressing patients had previously received trastuzumab, a HER2-directed monoclonal antibody, as their standard-of-care chemotherapy regimen, suggesting that prior trastuzumab therapy may sensitize tumor cells to cell-mediated lysis triggered by GP2. These results provide the insights, for further phase III trials on GP2 administration, that the breast cancer subset which GP2 would benefit most is the HER2-overexpressing subtype, and that TNBC patients should be excluded from further trials examining GP2.

Nelipepimut-S (NeuVax)

Nelipepimut-S (NPS, E75) is an MHC class I peptide derived from the extracellular domain of HER2 (amino acids 369–377) as an immunodominant epitope, combined with GM-CSF as a vaccine adjuvant (Neuvax). The safety and efficacy of Neuvax were previously shown in preclinical and phase I and II studies on HLA-A2, -A3, -A24, or -A26 positive patients with any level of HER2 expression [81,82]; however, due to a more intense immunologic response in the HER2-low expressing subset, a phase III trial of Neuvax monotherapy in this subset of patients was initiated and further terminated due to a lack of clinical benefit [83]. Evidence of potential immunologic synergy had been observed with trastuzumab in HER2-low expressing tumors in preclinic through increasing immune cell infiltration and the trastuzumab-mediated facilitation of HER2-Ag presentation by dendritic cells, which causes a subsequent cytotoxic T-cell response [84,85]. Therefore, a phase II randomized trial of Neuvax plus trastuzumab in HER2-low expressing, node-positive and/or TNBC patients (NCT01570036) to prevent recurrences was initiated. In this trial, disease-free patients were randomized to receive one year of trastuzumab per standard indication in combination with Neuvax (vaccine group) or GM-CSF (placebo group) every 3 weeks for 6 doses, starting with the third dose of trastuzumab. Patients received four booster inoculations every 6 months after completing the primary vaccine series. The interim analysis of this trial proved the vaccine to be safe, with no statistically significant difference in the observed toxicities between the arms and no grade 4/5 toxicities. After about 19 months of follow-up, TNBC patients demonstrated a statistically significant improvement in DFS compared to the placebo arm (*p* = 0.02) [37]. A further study on the correlation between HLA types and responses proved that all evaluated class I HLA types (A2, A3, A24 and A26) benefited from vaccination (HR = 0.29), especially HLA-A24+ patients (HR = 0.08, *p* = 0.02), despite having the lowest binding potential to E75 among HLA subtypes, which can be attributed to the lower Ag exposure and subsequent lower risk of tolerance seen with low-affinity peptides [35]. In a planned exploratory analysis of the TNBC subgroup of patients, a significant clinical benefit was observed in DFS after 26.1 months of follow-up (*p* = 0.013), in contrast to patients with hormone-receptor positive, node-positive and node-negative diseases, with which the vaccine proved to have no significant clinical benefit [34,36]. Also, subgroup analysis of the TNBC cohort revealed a greater benefit in patients who had received neoadjuvant chemotherapy, had lower HER2 expression (IHC 1+), were HLA-A24 positive, were over 51 years of age, or had early-stage disease (I/II according to AJCC 7th edition), providing insights for selecting ideal target populations for further trials to derive the most benefit from Neuvax [33,38]. Overall, these promising efficacy results, along with the reasonable safety of this treatment approach, i.e., Neuvax plus trastuzumab, present a potentially effective treatment approach for TNBC patients, which needs to be further evaluated in a phase III trial.

##### 3.1.1.2. Wilms Tumor 1 (WT1)

Galinpepimut-S

The Wilms tumor 1 gene, which was originally demonstrated to be involved in Wilms tumors in children [86], is an oncogene proven to be overexpressed in a number of hematologic and solid malignancies [87,88,89]. This gene is also expressed by normal mesodermal tissues, and translated into the zinc finger transcription factor WT1, which is involved in numerous critical cellular processes, including apoptosis and differentiation [90,91]. WT1 is one of the most studied immunotherapeutic targets in cancer, and hence, WT1-based peptide vaccines have been developed and evaluated in clinic with evidence of their potential safety and efficacy [92,93].

Galinpepimut-S (GPS) is a multi-peptide vaccine produced through the mixture of four WT1-analog peptides, two of which are synthetic (heteroclitic short (WT1-A1) and long peptides, which trigger CD8+ and CD4+ plus CD8+ immune responses, respectively) and the two remainder (331 and 427) native, triggering CD4+ responses [94]. These peptides encompass over 20 epitopes of the wild-type WT1 protein, thus triggering strong immune responses against multiple types of WT1-expressing cancers in patients with different HLA types. Several clinical trials have been performed and are ongoing with GPS as an immunotherapeutic, mainly in malignancies of myeloid origin, such as AML and MDS [95]. An actively recruiting phase 1/2, non-randomized trial evaluating the safety and efficacy of the use of concurrent GPS and pembrolizumab in up to 90 patients with selected advanced cancers, including AML and ovarian, colorectal, small-cell lung, and triple negative breast cancer (second line), is now ongoing (NCT03761914).

##### 3.1.1.3. Mucin 1 (MUC1)

Tecemotide (Stimuvax, L-BLP25, Emepepimut-S)

Mucin 1 (MUC1), a well-characterized TAA, is a transmembrane glycoprotein with normal expression on epithelial tissues which acts as a hydrator and moisturizer as well as a protective barrier on the cell surface, but is overexpressed and aberrantly glycosylated in malignantly transformed cells, and promotes the development and migration of cancer cells via participating in intracellular signaling pathways [96]. Several MUC1-based immunotherapeutic approaches exist for MUC1-expressing solid tumors, including targeted antibodies and vaccines [97,98]. Tecemotide (Stimuvax, L-BLP25) is a MUC1-based, lipopeptide vaccine which is actually a synthetic peptide of the tandem repeat region of the MUC1 protein backbone (BLP25), encapsulated in liposomes (L-BLP25). The liposome-based formulation allows for a more robust T-cell and humoral immune response to BLP-25 [99]. Tecemotide has entered numerous phase II and III trials in several indications, originally in non-small cell lung cancer [100]. Since MUC1 is overexpressed in about 90% of breast carcinomas [101], its targeting may present a promising therapeutic approach for breast cancer. In this regard, Singer et al. performed the phase II, ABCSG trial (EudraCT#2011-004822-85) to evaluate the addition of tecemotide to SoC, neoadjuvant chemotherapy in HER2-negative, early invasive breast cancer patients. In this multicenter trial, postmenopausal, ER+ breast cancer patients were randomized to receive either aromatase inhibitor alone (CG) or in combination with tecemotide (VG); premenopausal, TNBC, or ER− subtypes received either anthracycline-and-taxane-based chemotherapy alone (CG) or combined with tecemotide (VG). In the whole study population, as well as in the TNBC subgroup, no statistically significant difference was observed in the primary (Residual Cancer Burden, RCB) and secondary (pathologic Complete Response, pCR) objectives after about 29 weeks of follow-up. However, the vaccine proved to be safe, as evidenced by the absence of significant differences in the occurrence of AEs between VGs and CGs [39]. Taken together, these results suggest that tecemotide might not be an ideal vaccine candidate for breast cancer patients, and for TNBC patients in particular; however, further studies of this vaccine in the setting of recurrence prevention in longer follow-up durations might present better results.

Investigational MUC1 Vaccine

Another MUC1-based vaccine for stage I-III TNBC patients has been proposed by researchers of the Case Comprehensive Cancer Center, in which Poly-ICLC (Hiltonol) is used as an adjuvant. The safety and efficacy of this vaccine in mounting immune responses has been investigated in an early phase I trial (NCT00986609). In this study, 29 TNBC patients receive four monthly subcutaneous inoculations of the vaccine, followed by booster vaccinations on weeks 52 and 56, and are monitored for the occurrence of serious adverse events as well as anti-MUC1 antibody responses. Although the trial is claimed to be completed, no results have yet been posted.

##### 3.1.1.4. Globo H

Adagloxad Simolenin (AS/OBI-822)/OBI-821 (Globo H-KLH)

Globo-H is a tumor-associated carbohydrate Ag (TACA) which is structurally ceramide-linked, and is expressed in many tissues, including breast, prostate, and pancreas tissues, with a limited pattern [102]. Given the important immunosuppressive and angiogenic potentials of Globo-H [103,104], it has been proposed as a vaccine candidate and evaluated in clinic in phase I trials of patients with prostate and breast cancers [105,106]. Since TACAs are unable to provoke T-cell immune responses, the conjugation of Globo H to immunogenic moieties, such as keyhole limpet hemocyanin (KLH), is necessary in order to design an effective cancer vaccine. This carrier protein helps trigger both T- and B- cell responses to the whole conjugate, and, when combined with the adjuvant QS-21, strong Globo-H-specific immune responses are elicited [105]. In a large randomized, multicenter phase II trial of AS/OBI-821 in metastatic breast cancer patients (NCT01516307), 349 patients were recruited in total, 45 of whom had TNBC. After a median follow-up duration of about 22 months, no significant improvement in the primary outcome of the study (PFS) was detected, which was also the case for the study subgroups, including TNBC. However, a detectable humoral response was observed through the elevation of serum IgM and IgG levels against Globo-H, which significantly correlated with PFS in patients with IgG levels above 1:160. This IgG rise was more significant in patients without a progressive disease; thus, further phase III trials of patients with an early-stage disease without heavy pretreatment might elucidate more significant results. The vaccine also proved to be safe with most AEs relating to injection site reactions, fevers, or grade 1/2 systemic AEs. Another important finding was that patients completing all nine planned inoculations had a better, albeit non-significant PFS (*p* = 0.06); therefore, continuous vaccination might possibly improve survival [40]. Considering these findings, a phase III trial of AS/OBI-821 versus SoC in non-metastatic, early-stage TNBC patients (GLORIA study) is ongoing (NCT03562637), extending vaccine inoculations to 21 injections over 100 weeks to assess 5-year DFS and other efficacy and safety indices [107,108].

##### 3.1.1.5. New York Esophageal Squamous Cell Carcinoma-1 (NY-ESO-1)

CDX-1401

NY-ESO-1 is a cancer testis Ag with a limited expression pattern in normal tissues, but its re-expression has been observed in several solid tumor types, including breast cancer. Nevertheless, NY-ESO-1 has been demonstrated to be highly immunogenic, since it can spontaneously induce specific humoral and cellular responses resulting in tumor regression [109]. Thus, it has been considered as a potential target for immunotherapy in several solid tumor types. To foster the immune response to this Ag as a potential vaccine candidate, a peptide consisting of a fully human monoclonal antibody against the dendritic cell receptor decalectin (DEC-205, CD205) fused to NY-ESO-1, named CDX-1401, has been developed, which is able to bind DEC-205 on the surface of dendritic cells, thus promoting Ag uptake and presentation by these cells and inducing robust anti-NY-ESO-1 responses. CDX-1401 combined with the Flt3 ligand CDX-301 and Hiltonol has demonstrated safety and signs of clinical efficacy in phase I/II trials on patients with melanoma [110,111]. Accordingly, in a phase I/II trial of combined subcutaneous triweekly doses of CDX-1401, along with Hiltonol and pembrolizumab in patients with selected NY-ESO-1-positive advanced malignancies, including TNBC, the safety and tolerability of this regimen, along with its ability to induce anti-tumor responses and clinical efficacy indices (including ORR, median time to tumor response, and median PFS/OS), were evaluated (NCT02661100). However, the study was withdrawn without enrolling any participant in 2016 due to the “drug being unavailable.”

##### 3.1.1.6. Survivn

Helper/Killer Hybrid Epitope Long Peptide (H/K-HELP)

In order to evoke a complete immune response by inducing both Th_1_ cells and CTLs to better overcome the immunosuppressive TME, Nishimura et al. developed synthetic long peptides composed of both helper- and killer- T cell immunogenic epitopes of several different TAAs, brought together by means of a glycine linker. Such long peptides have been proven to be superior to short MHC-binding peptides at inducing cytotoxic responses and circumventing possible immune tolerance mechanisms [112]. Accordingly, in a patient with colon cancer treated with a MAGE-A4-associated helper/killer hybrid vaccine, Th_1_-dependent cellular and humoral anti-tumor responses were observed, without the occurrence of SAEs, except for a grade 2 injection site reaction. The patient also experienced a significant decrease in tumor growth and the level of the carcinoembryonic Ag (CEA) tumor marker and reached stable disease [113]. Further, in a phase I study of two different H/K-HELP vaccines of MAGE-A4 and Survivin on patients with solid tumors, complete regression of cervical node recurrence was observed in a chemo-resistant TNBC patient four weeks after the administration of one cycle of Survivn H/K-HELP vaccine [44]. While this is an interesting vaccination approach, no further trials of this vaccine on patients with solid tumors have been recorded.

##### 3.1.1.7. GD2/LeY

P10s-PADRE

As a means of augmenting immune responses to TACAs, pan-immunogenic carbohydrate-mimetic peptides (CMPs) have been developed which are able to provoke both humoral and cellular immune responses to the TACAs they mimic [114]. CMPs present interesting opportunities for active cancer immunotherapy, since many TACAs are involved in cancer invasion and metastasis. P10s in one of these CMPs which mimics the TACAs Lewis Y (LeY) and GD2 has been shown to induce cellular and humoral immune responses and inhibit tumor growth in mice [115]. P10s was conjugated to the pan-T cell epitope PADRE along with the Montanide ISA 51 adjuvant to develop the cancer vaccine P10s-PADRE, and the tolerability and immune response to this vaccine was evaluated in stage IV breast cancer patients in an early phase I study. The vaccine was safe and well-tolerated, and effectively induced Ag-specific humoral responses along with signs of clinical benefit [116]. Accordingly, a phase I/II study of P10s-PADRE is currently recruiting TNBC patients with stage I, II, or III disease, which evaluates the combination of vaccine and standard-of-care neoadjuvant chemotherapy against chemotherapy alone (NCT02938442). Patients will receive three weekly inoculations prior to neoadjuvant chemotherapy. The main objectives of this trial include safety and the demonstration of clinical response assessed via pCR. The study is expected to be completed by 2024.

##### 3.1.1.8. NGcGM3

NGcGM3 /VSSP

NGcGM3 is the N-glycosylated form of the ganglioside GM3, which is overexpressed in a variety of tumor tissues including breast tumors, but not in normal cells. Besides being highly tumor-specific, NGcGM3 is known to harbor immune-suppressive activities through down-regulation of CD4 Ag [117], and is therefore considered to be an ideal target for cancer immunotherapy [118]. Since glycolipids are poorly immunogenic, as is the case for TACAs, NGcGM3 has been conjugated via anionic detergents to the outer membrane protein complex of *Neisseria meningitidis*, forming very small-sized proteo-liposomes (VSSPs) to overcome the immune tolerance to this Ag [118]. The NGcGM3/VSSP vaccine has been evaluated in numerous trials on patients with melanoma, breast, and non-small cell lung cancers, and has demonstrated promising results in terms of safety, induction of humoral response, and improvement of OS in metastatic breast cancer patients, especially in patients with non-visceral metastasis [119,120]. Following these promising results, the combination of this vaccine with nimotuzumab, a humanized anti-EGFR mAb, is being evaluated in a phase I/II trial on patients with metastatic TNBC and non-small cell lung cancer (RPCEC00000218 and RPCEC00000270, respectively, Cuban Public Registry of Clinical Trials), with the primary outcome of OS, and secondary outcomes of safety, OR, PFS, QoL, and immune response.

##### 3.1.1.9. Folate Receptor-α (FR-α)

Folate is an important factor involved in several metabolic pathways, including the synthesis of amino acids and nucleotides. The membrane receptor of folate, namely FR-α, has been seen to be overexpressed in a number of cancers, including TNBC, due to its role in the overgrowth of tumor cells [121]. It has been reported that over 70% of TNBC patients express FR-α to some degree [122]. Thus, active immunotherapy with FR-α epitopes might present attractive vaccine design approaches. Accordingly, two phase II clinical trials of multi-epitope FR-α vaccines in the adjuvant setting for TNBC patients have been registered (NCT02593227 and NCT03012100, with 80 and 280 patients being enrolled, respectively). Both trials are evaluating vaccine formulations in combination with GM-CSF as a vaccine adjuvant and cyclophosphamide as an immune priming regimen, and their objectives include safety, vaccine-specific immune responses, and survival indices (overall, disease-free and relapse-free survival). None of the results of either of the trials have been posted so far.

##### 3.1.1.10. α-Lactalbumin (α-La)

α-Lactalbumin (α-La) is a differentiation Ag which is normally expressed on breast tissue during late pregnancy and lactation, but is also overexpressed in malignantly transformed cells of the breast in the TNBC subtype [123]. Preclinical evidence has suggested α-La-based vaccines to be of benefit for TNBC prophylaxis without inducing any autoimmune inflammation, since α-La expression in healthy individuals is confined to their lactation period [124]. Moreover, the stable expression of α-La during tumor development in TNBC-bearing mice as well as the induction of inflammatory T-cell responses in human PBMCs primed with recombinant α-La [125] suggest α-La as a suitable therapeutic cancer vaccine candidate. Accordingly, a phase I dose-escalation trial of 24 non-metastatic TNBC patients is ongoing, where the α-La-vaccine as well as zymosan as an adjuvant are administered biweekly for 3 successively higher doses of the vaccine (10 to 1000 µg) to determine its safety, efficacy in inducing immunologic responses, and maximum tolerated dose [126]. If the results demonstrate signs of benefit, this vaccine could become one of the first of its kind for preventing TNBC development in high-risk individuals, such as those with *BRCA* mutations.

##### 3.1.1.11. Multi-Peptide Vaccines

XBP1 , CD138, and CS-1 Vaccine: PVX-410 (PVX, OncoPep)

PVX-410 is an HLA-A2 restricted, tetrapeptide vaccine which includes two splice variants of the XBP1 transcription factor along with CD138 and CS-1, all of which are overexpressed TAAs in TNBC. Previous phase I studies of this investigational vaccine candidate have demonstrated its safety and ability to induce immune responses in multiple myeloma patients [127]. In a subsequent phase I trial of PVX-410 combined with durvalumab, 22 HLA-A2+, stage II/III TNBC patients were recruited. Patients received six fortnightly doses of PVX-410, co-administered with Montanide and Hiltonol as vaccine adjuvants, and two doses of durvalumab (1500 mg) at the fourth and sixth vaccine inoculations [128]. After a median follow-up of 15.4 months, the vaccine turned out to be well-tolerated with mainly injection site reactions and fatigue, few grade 3 AEs, and no grade 4/5 events. The immune response evaluation revealed durable PVX-specific T-cell responses in almost all patients which were maintained for up to 6 months, and an 18% recurrence and 9% death events were observed in the whole population [41]. Subsequently, another phase I trial investigating the safety, immune response, and efficacy of concurrent PVX-410 and pembrolizumab in 19 HLA-A2+ metastatic TNBC patients followed. After a median follow-up of 36.8 months, the median OS and PFS were 19.9 and 2.3 months, respectively. Stable disease was reached in 47% of patients, while no patients experienced complete/partial responses. Regarding safety, the most common adverse events, including fatigue and injection site reactions, were of grade 2, and few grade 3 and 4 AEs occurred were attributable to pembrolizumab. Moreover, specific cytotoxic T-cells responses and an increase in specific memory T-cell were observed in the majority of patients [42]. The data of both trials are promising, and hence, further phase II trials of this vaccine are anticipated.

Multi-Peptide Active Immunotherapy

In a pilot study of 25 patients with refractory cancers, including high-grade ovarian cancer, soft sarcomas, pancreatic cancer, and TNBC, the approach of combining immunogenic chemotherapy and multi-peptide active immunotherapy was adopted in order to revert the so-called “chemoresistance” of these patients. To this end, low-dose doxorubicin and oxaliplatin were administered every two weeks, along with weekly, subcutaneous inoculations of a combination of 22 peptides in inguinal and axillary lymph nodes followed by local injections in areas with high tumor activity (according to CT/PET scan). This peptide pool contained proteins involved in cancer development and migration, such as Fascin, Ape-1, Bcl-2, and VCP. The treatment regimen lasted for 4 weeks, and caused clinical remission (according to CT scan) which correlated with CD8 infiltration in the injection sites in all patients. Moreover, the treatment was well-tolerated with minimal local events in injection sites [51]. The combination of low-dose chemotherapy alongside active immunotherapy seems to hold numerous potential benefits for patients with refractory cancers. While no other details or updates of the trial have been published, these promising results suggest this combination should be further evaluated in larger patient populations.

#### 3.1.2. Individualized Peptide Vaccines

##### 3.1.2.1. Personalized Peptide Vaccination (PPV)

Another approach to the development of personalized vaccines for cancer patients is to select a combination of TAA-derived peptides to which cellular or humoral immunity is present before inoculation in each individual. With the presence of the pre-existing memory of cytotoxic T-lymphocytes (CTLs), these cells are already primed against the desired TAAs, and hence, vaccination can induce prompt and strong antitumor responses. In an early study on patients with recurrent gynecological cancers, 10 HLA-A2/A-24-positive patients were vaccinated with a maximum of four TAAs to which Ag-specific CTLs were present pre-vaccination in their peripheral blood samples. While adverse events were mainly restricted to grade 1, injection site reactions, three of the five patients with cervical cancer experienced objective tumor regression [129]. Extending this approach to several other advanced cancers including melanoma [130], glioma and glioblastoma [131,132], renal cell carcinoma [133], and colorectal [134], prostate [135], lung [136], and gastric [137] carcinomas in phase I trials, vaccination turned out to be safe and well-tolerated along with signs of clinical benefit through lowering the level of cancer-associated biomarkers, maintaining stable disease, inducing tumor regression, or increasing the survival rates. Although both cellular and humoral antitumor immune responses were observed in the majority of patients in each trial, further investigation demonstrated a clinically meaningful correlation of post-vaccination peptide-specific IgG levels with overall survival, while such correlation was weaker in cellular immune responses [138,139].

Favorable responses of the aforementioned phase I trials prompted the researchers to evaluate the clinical benefit of PPV in phase II studies through mono or combination therapy [140,141]. Accordingly, a phase II study on patients with metastatic breast cancer who had failed standard chemotherapy was conducted, where 31 candidate peptides were selected from a pool of multiple TAAs, from which up to 4 HLA-IA-binding peptides (A2, A3 family, A24, and A26) inducing the highest Ag-specific IgG responses in each individual were chosen as a personalized vaccine candidate and administered in combination with their conventional chemo- or endocrine therapy (UMIN000001844). In the early reports of this trial, patients receiving this vaccine for two cycles (including six inoculations every 1–2 weeks) demonstrated notable elevations in peptide-specific IgG responses and signs of clinical benefit, without any significant differences between patients with triple-negative subtypes and other cases. Moreover, no severe adverse events were observed in these patients [46,47,48].

According to their most recent report, 79 breast cancer patients were recruited in this trial, of which 18 were of triple-negative subtype. Two patients with TNBC experienced significant clinical benefits: one was a complete responder and another demonstrated partial response. In total, the median PFS and OS for TNBC patients were 7.5 and 11.1 months, respectively, which seems encouraging. Of note, a record of more than four previous lines of chemotherapy was significantly associated with a poorer prognosis in patients receiving PPV, and thus, the authors do not recommend PPV vaccination for breast cancer patients with more than three lines of previous chemotherapy. Regarding safety, all patients in the trial demonstrated grade 1/2, but not 3/4, injection site reactions. Moreover, other grade 3/4 adverse events, which occurred in 52% of patients, were strongly associated with concurrent chemotherapy and disease progression, suggesting PPV as a safe immunotherapeutic approach [45]. Taken together, PPV might present important therapeutic potentials for TNBC patients; however, further phase III trials of greater patient populations investigating the efficacy of PPV alone or combined with chemotherapy or other immunotherapeutic agents are awaited.

##### 3.1.2.2. KRM-19

While PPV demonstrated clinical benefits in patients with advanced cancers, these benefits could not sufficiently warrant the approval of PPV for these patients. Since this could be attributed to the low induction of the immune response by only a few peptides present in the formulation of PPV, the same research team focused on developing a novel vaccine formulation consisting of 20 peptides from 12 different TAAs for castration-resistant prostate cancer patients with different HLA alleles. This vaccine, namely KRM-20, was demonstrated to be safe and effective in mounting peptide-specific CTL and IgG responses in a phase I trial [142]. Based on these encouraging results, 19 peptides of 11 TAAs, including SART3, HNRPL, and WHSC2, were selected from the previously reported pool of 31 peptides according to their immunogenicity and safety for patients with metastatic TNBC and formulated as KRM-19. Accordingly, 6 weekly inoculations of KRM-19 with the dose of 19 mg/mL were administered to 14 HLA- A2, A3, A11, A24, A26, A31, or A33-positive breast cancer patients in a phase II study (UMIN000014616). In its primary report, a 12.5% objective response rate (ORR) and 62.5% clinical benefit rate (CBR) was observed in 10 patients who completed the vaccination series, along with the adverse events of mainly grade 1 and 2 injection site reactions [50]. While the median OS was 11.5 months in all 14 patients of the study, those completing the whole 6 inoculations (10 patients) experienced a median OS of 24.4 months. As expected, KRM-19 induced potent IgG responses that were significantly higher than the immune boosting previously observed with the 4-peptide PPV. Rapid disease progression, which was observed in four patients who could not finish vaccination series, was linked to high C-reactive protein levels or more than three previous systemic chemotherapies. While five cases of grade 3 and two cases of grade 5 AEs were observed, all such events were related to disease progression, and only injection site reactions were correlated to the vaccine [49]. Although the number of patients in this study is rather small to elucidate the exact efficacy and safety profile of KRM-19, these encouraging results warrant further phase II trials with larger populations of TNBC patients with respect to subpopulations who are probably better candidates for treatment with this vaccine.

##### 3.1.2.3. PepIVAC-01

Another approach to the development of personalized vaccines is to thoroughly analyze the whole exome, transcriptome, and HLA-ligandome of the autologous tumor tissue of each individual to determine the most ideal immunological targets for vaccine development. This approach has been adopted by Haen and his colleagues, where an individualized multi-peptide vaccine was developed with Montanide ISA 51 as an adjuvant and administered in a pilot trial of patients with several types of advanced cancers, including non-small cell lung cancer, cholangiocarcinoma, sarcoma, and TNBC. In this three-tier escalation trial, the vaccine combined with topical imiquimod is administered as the first step, followed by a toll-like receptor ligand (XS-15) as a lipopeptide adjuvant and a checkpoint inhibitor. The primary focus of this trial is the safety and feasibility of this personalized vaccine-based approach, with its secondary objective being the monitoring of the induced immunological responses of patients [143]. Since this approach involves both active and passive immunotherapeutic approaches, which might prove to be superior to the vaccine-only strategies discussed above, the results of this trial are eagerly awaited.

##### 3.1.2.4. NeoAg Long Peptide Vaccine

A major problem in the immunotherapy of cancer is that the immunogenicity of “shared” TAAs is not strong enough to mount effective antitumor responses due to immune tolerance mechanisms, reinforcing tumor immune escape. NeoAgs, which arise as a result of numerous mutations in tumor cells, open up new opportunities for specific and strong targeting of cancer cells, since they are both cancer cell-specific and highly immunogenic due to not being prone to central tolerance [144]. Targeting neoAgs in TNBC, a tumor known to harbor a large number of somatic mutations [145], could be a potentially successful approach for developing cancer vaccines. Since neoAgs are greatly distinct for each individual, they are identified through next-generation sequencing (NGS) technologies and multiple bioinformatics tools to predict the epitopes with the highest immunogenicity and expression among tumor cells.

Accordingly, an individualized synthetic long peptide neoAg vaccine with poly-ICLC (Hiltonol) as an adjuvant was developed for TNBC patients, with each vaccine formulation being produced individually based on the NGS of tumor biopsies. The safety and immunogenicity of this vaccine was being evaluated in a phase I trial on 15 TNBC patients who had not achieved a pCR after neoadjuvant chemotherapy and are thus at high risk for recurrence (NCT02427581); however, the trial has been recently withdrawn due to the “drug not being available.” Since, on a theoretical basis, the combination of neoAg vaccines and other chemo/immunotherapeutic agents can lead to better effects through priming immune responses and ameliorating the immunosuppressive tumor microenvironment, a randomized phase II study of this vaccine formulation along with chemo- (nab-paclitaxel) and checkpoint inhibition- (durvalumab/tremelimumab) therapy, with an initial 18-week run-in of gemcitabine and carboplatin, is currently recruiting metastatic TNBC patients (NCT03606967). The primary objective of the trial is PFS; the secondary objectives include safety, ORR, CBR, OS, and immune response [146].

### 3.2. Cell-Based Vaccines

#### 3.2.1. Dendritic Cell (DC) Vaccines

Dendritic cells are potent Ag-presenting cells (APCs) priming anti-tumor T-cell responses which can be autologously isolated from the peripheral blood, stimulated with specific cytokines, pulsed with tumor antigens, Ag-encoding DNA/RNAs, or tumor cell lysates ex vivo, and administered back to patients as active immunotherapeutic agents, or cell-based vaccines [147]. Alternatively, DC-vaccines can be manufactured through inducing differentiation in peripheral blood-derived monocytes or hematopoietic stem cells via specific cytokines [148]. DC cancer vaccines have achieved success in a number of trials; however, a lack of clear clinical benefits in most of the trials has hampered their extensive use. Such a deficit has been mainly attributed to the immunosuppressive TME, reinforcing tumor cell evasion and dampening DC function. Thus, a combination of DC vaccines with other therapies targeting the immunosuppression associated with the TME, e.g., immune checkpoint inhibitors, might help boost their anti-tumor potentials [149]. Furthermore, chemo/radiotherapy can help boost their efficacy through inducing apoptosis in tumor cells and enhancing the release of their Ags, thus priming the administered DCs against tumor cells. DC vaccines are most commonly administered intradermally, but subcutaneous or intratumoral injections have also been reported.

##### 3.2.1.1. Whole Cell-Pulsed DC Vaccines

Tumor Lysate-Loaded DC Vaccine

In a non-randomized, phase II trial, the addition of a monocyte-derived, autologous tumor-lysate pulsed DC vaccine to neoadjuvant chemotherapy (NAC) was evaluated in 39 treatment-naïve patients with stage I-III HER2-negative BC, of whom 36% were of the TNBC subtype. The primary outcome measure was pCR, which was significantly higher in VG (26.3%) compared to a historic cohort of 44 patients treated with NAC alone as the CG (9.09%) after 7.5 years of follow-up. Additionally, no grade ≥ 3 vaccine-related AEs were observed [53]. In a further record, pCR rate was reported to be 17% vs. 67% in CG and VG of the TNBC sub-population, and significantly increased CD8-TIL levels were also observed in TNBC patients not reaching pCR [54]. Additionally, a further immune response evaluation of the patients before and after treatment revealed an increase in both humoral and cellular responses and NK cells, along with decreases in the population of myeloid-derived suppressor cells, and the PD1 and TIM3 immune checkpoints of CTLs [150]. According to a more up-to-date report of the trial, in which the study objectives were expressed as being stratified according to their PD-L1 status, vaccine treatment was able to produce a more significant response in terms of enhancing pCR in patients with PD-L1-negative tumors, in whom a less immunosuppressed TME is anticipated [151]. In their final report after a median follow-up period of 8 years, pCR was significantly improved in the overall population, with the TNBC subpopulation experiencing the highest pCR among other subtypes (50% in the VG vs. 30.7% in the CG, *p* = 0.25); albeit the benefit in this subpopulation was non-significant. Such an inability to discern a significant difference can be in part attributed to the low sample size of the TNBC subgroup, which included only a total of 30 patients in both arms. The AEs were mild and mostly confined to injection site reactions, and the occurrence of grade ≥3 Aes was similar between the two groups [52]. Although this trial could not demonstrate a gain of benefit in either event-free or overall survival, these results suggest that appropriate patient selection based on the expression of immune checkpoints and biological subtypes in early BC patients might help elucidate the clinical benefits of DCV. Hence, larger randomized phase II/III studies of a combination of NAC and DCV are anticipated.

Heat-Induced Apoptotic Tumor Cell-Loaded DC Vaccine

The safety and efficacy of a DC vaccine loaded with Ags of heat-shock-induced apoptotic tumor cells in TNBC patients was evaluated in a randomized, multi-center trial in three Chinese hospitals. In total, 168 TNBC patients were recruited, of whom 112 and 56 were assigned to vaccine and control groups, respectively. The primary outcomes were disease progression time (DPT) and progression-free survival (PFS), and the secondary outcomes were safety and immune responses. After three cycles of vaccination and a two-year follow-up period, a significant increase in both primary measures of outcome, DPT and PFS, was observed, as well as the notable induction of tumor-specific CD8 responses. The vaccine was also safe and well-tolerated [152]. Further follow-up results of this trial on larger populations are anticipated.

##### 3.2.1.2. Ag-Pulsed DC Vaccines

Cyclin B1/WT1/CEF-Pulsed DC Vaccine

Another study of cell-based vaccines in TNBC patients evaluated the efficacy of an Ag-loaded DC vaccine combined with chemo- and radio- therapy. Monocyte-derived DCs were pulsed with cyclin B1 and WT1 as TAAs in TNBC, as well as the control viral Ag CEF, and activated with LPS, Clo75, and CD40 ligand. Ten patients with locally advanced TNBC received SoC NAC, combined with four IT and SC doses of the vaccine prior to surgery and three doses post-surgery. The primary and secondary outcomes were safety and pCR in the breast and axilla, respectively [57]. At the time of definitive surgery, four patients achieved a pCR, and three had minimal residual disease (RCB score = 1), making up a 70% combined pCR and RCB-1 rate. Three other patients had macroscopic residual disease in the breast and axillary lymph nodes, and grade 1/2 injection site reactions were observed in nine of the patients. Importantly, IFNγ-ELISPOT assays revealed significant Ag-specific immune responses post-treatment [56]. These results suggest this vaccine-based strategy to be safe with a promising response rate, and further evaluations of larger patient populations are warranted.

HER2 /HER3-Pulsed DC Vaccine

An actively recruiting phase IIa study of a HER2/HER3-targeting DC vaccine combined with pembrolizumab for the treatment of brain metastases from TNBC has been initiated since April 2022. In this non-randomized study, stage IV TNBC patients with measurable brain disease receive three triweekly doses of an HER2/HER3-targeted DC vaccine intradermally as well as pembrolizumab in the treatment phase, followed by maintenance doses of triweekly pembrolizumab until disease progression. The primary outcome measure is the central nervous system (CNS) objective response rate (ORR). The study is anticipated to be completed by 2025.

NeoAg-Pulsed DC Vaccines

By analyzing the tumor transcriptome and identifying neoAgs arising from non-synonymous mutations, personalized cancer targets can be recognized, leading to the development of highly efficient treatment strategies. Accordingly, in a phase I study of patients with TNBC who have already completed their SoC, DC vaccines pulsed with synthetic neoAg peptides were developed on an individual basis and administered intradermally to patients in six doses (NCT04105582/NCT04879888). The primary objective of the trial is safety, and the secondary objective is the immunogenicity of the vaccine as assessed via IFNγ-ELISPOT. None of the results of the trial have been posted to date.

### 3.3. Nucleic Acid-Based Vaccines

As a major advantage, nucleic acid-based vaccines are able to deliver a set of tumor Ags to the APCs, and are thus considered to be emerging platforms for novel multi-(neo)Ag, personalized cancer vaccines. Although few clinical studies have evaluated the feasibility of nucleic acid-based vaccines, preclinical studies are largely focusing on evaluating their efficacy in a multitude of tumor models. Furthermore, with the recent approval of two RNA-based COVID-19 vaccines, it is anticipated that nucleic acid-based vaccines will occupy a large share in future markets of cancer therapeutics.

#### 3.3.1. RNA Vaccines

RNA vaccines are synthetic mRNAs encoding one or more immunogenic Ags which are translated to their associated Ag upon uptake by APCs. Able to deliver multiple Ags, these platforms can better overcome tumor immune escape than single-Ag cancer vaccines. While they can be synthetized in a feasible manner in vitro, the issues of mRNA stability and delivering the RNA have been important challenges in their development. Various strategies have been adopted to overcome these issues, including considerations in the design of 5’ cap and poly-A tails, increasing their purity through efficient chromatography methods, and the use of highly efficient vectors for their delivery [15]. Lipid nanoparticle (LNP) carriers have been the most popular vector for mRNA vaccines; however, viral vectors and cell-based delivery platforms have also been evaluated [153]. The formulation may be delivered through intramuscular (IM), intradermal (ID), or subcutaneous (SC) routes.

##### 3.3.1.1. Autogene Cevumeran (RO7198457)

RO7198457 is an RNA-lipoplex vaccine designed individually based on neoAgs arising from each patient’s selected somatic mutations, also known as individualized NeoAg Specific immunotherapy (iNEST). The RNA-lipoplex formulation encodes for up to 20 neoepitopes selected “per patient,” which, upon translation, mount T-cell responses via presentation through APCs. Moreover, since RNA is an intrinsic ligand of TLR7/8, it can induce innate immune responses as well. Based on promising preclinical results of combining RO7198457 with checkpoint inhibition therapy, the combination of this vaccine with atezolizumab was evaluated in a phase I study of RO7198457 in patients with selected advanced/recurrent solid tumors. In the dose-escalation phase, multiple intravenous doses of the vaccine along with atezolizumab were evaluated in 29 patients [154]. In the dose-expansion phase, the safety of three selected doses of the vaccine and its efficacy in mounting specific immune responses were evaluated in multiple cohorts of 132 patients, including in 24 checkpoint-inhibitor naïve TNBC patients. Patients were administered with nine weekly/biweekly doses of the vaccine plus 1200 mg of atezolizumab every 3 weeks in the induction phase. In the maintenance phase, the vaccine was injected every 24 weeks along with atezolizumab every 3 weeks until disease progression occurred. The vaccine was able to effectively induce pro-inflammatory cytokines, along with tumor-specific T-cell responses in 73% of patients, and signs of neoepitope-specific T-cell infiltration in tumor sites. The ORR in the TNBC cohort was 4%. The combination was well-tolerated with no DLTs observed, and had low-grade, transient systemic side effects [58]. The combination of RO7198457 with pembrolizumab as first-line treatment of patients with melanoma (NCT03815058), and with atezolizumab as adjuvant treatment in NSCLC patients (NCT04267237), is currently being investigated in two randomized phase II studies.

##### 3.3.1.2. Mutanome-Engineered RNA Immunotherapy (MERIT)

The MERIT project is a multi-center, individualized RNA vaccine therapy program for TNBC patients, targeting patient-specific tumor mutations. This personalized vaccination strategy is based on two steps: first, vaccines are formulated per patient from an off-the-shelf mRNA warehouse of shared TSAs in TNBC (MERIT WAREHOUSE). Next, according to tumor NGS profiling of each patient, non-synonymous mutations are identified, ranked by predicted immunogenicity, and synthesized as neoAg mRNAs on-demand (MERIT MUTANOME). The mRNAs are formulated as nano-lipoplexes and administered intravenously [155]. Through this approach, a wide range of tumor Ags, i.e., “tumor Agome” can be presented to the immune system. In the first in-human trial of MERIT in 2016, 30 TNBC patients were planned to be recruited after SoC therapy. Patients in arm 1 received eight vaccination cycles of a 2-3 mRNA-containing vaccine from the MERIT WAREHOUSE, based on RNA profiling of each patients’ tumor specimen. An extra eight cycles of the MERIT MUTANOME vaccine containing up to 20 neoepitopes was administered to patients in arm 2. Standard radiotherapy was continued for all patients. The aim of the study was to assess the feasibility, safety, and efficacy of the vaccine formulation. Based on the immunologic assessment data of 14 patients, all patients experienced polyepitope T-cell responses against 1–10 of the vaccine neoepitopes [156]. Other preliminary clinical data have also demonstrated the safety and feasibility of this approach [157]. The full study results are awaited.

#### 3.3.2. DNA Vaccines

Similar to RNA vaccines, DNA vaccines work by inducing immune responses to the specific TAAs or TSAs they encode. Once administered, the DNA enters the nucleus of the APCs and is transcribed to mRNA, from which the relevant Ag is translated. Compared to their RNA-based counterparts, DNA vaccines are more stable, and easier and more cost-effective to manufacture. Moreover, a single DNA molecule can generate several mRNA transcripts, which, upon translation, can induce both humoral and cellular responses. The DNA may be delivered through viral or plasmid vectors as naked or with LNPs or polymer-based nanoparticles, and administered via IM or ID electroporation techniques or mechanical gene guns to be directly transferred to the cells [158].

##### 3.3.2.1. Viral Vectors

While highly efficient in terms of gene delivery, safety hazards with the use of viral vectors have long been an issue of concern. Accordingly, replication-deficient viral vectors devoid of virulence factors have been developed, including genetically modified adeno/retroviral vectors, as well as the poxvirus Modified vaccinia virus Ankara (MVA), which have proved to be safe in many preclinical experiments and in the clinic [159].

Modified Vaccinia Virus Ankara (MVA)
BN-Brachyury-TRICOM

Brachyury, a transcription factor expressed in early embryonic development, is a well-known TAA involved in cancer progression and invasion in several epithelial tumors including breast cancer, as well as in the epithelial-to-mesenchymal-transition (EMT) process [160]. One of the major advantages of brachyury as a potential target for cancer immunotherapy is its almost-exclusive expression in tumor tissues [161]. Breast cancer of the TNBC subtype shows a significant brachyury expression profile compared to other subtypes, and its expression level has shown associations with the disease stage in TNBC [162]. Bavarian Nordic (BN)-brachyury-TRICOM is a recombinant vector vaccine expressing brachyury as well as the three T-cell co-stimulatory molecules B7.1, ICAM-1, and LFA-3. The vaccine follows a prime-boost strategy with two platforms: Modified Vaccinia Ankara (MVA)-brachyury-TRICOM virus as the priming vaccine, and fowlpox virus (FPV)-brachyury-TRICOM as the boosting vaccine [163]. A phase I trial with BN-brachyury-TRICOM of 63 patients with metastatic solid tumors revealed its safety, with adverse events restricted to grade 1/2 injection site reactions. The six-month PFS was 50%, and six patients reached stable disease. Of note, CEA- and MUC1-specific T-cell responses were observed in most patients as cascade Ags, suggesting potential tumor cell disruption [164]. The combination of BN-brachyury-TRICOM with other systemic therapies is being evaluated in several trials of patients with chordoma, prostate, and breast cancers [163]. In the BrEAsT study, three single-arm, phase Ib trials on patients with advanced-stage breast cancer study the combination of BN-brachyury along with T-DM1 (adotrastuzumab emtansine), entinostat (histon deacetylase inhibitor), and M7824 (Bintrafusp-α, bifunctional fusion protein targeting both TGF-β and PD-L1). This combination therapy has been shown to increase specific T-cell responses and enhance Ag presentation, while suppressing regulatory T-cells and myeloid-derived suppressor cells in pre-clinical models [165]. Based on histological subtypes, patients receive different vaccine and drug combinations. In arm 1, TNBC patients receive the vaccine along with M7824, while the combination of the vaccine with T-DM1, M7824, with or without entinostat, is being studied in HER2-positive patients in arms 2 and 3 of the study. Using a Simon 2-stage trial design, patients are accrued in a stepwise fashion while being closely monitored for any dose-limiting toxicities and responses to treatment. A maximum of 13 patients are expected to be recruited in this phase I trial on TNBC patients [165]. The trial is currently active and recruiting patients.

*p53MVA*

Tumor protein 53 (TP53, or p53), a well-known tumor suppressor, is a regulator of cell division, and its mutations are frequently observed in the majority of solid tumors. Of note, p53 mutations are observed in about 80% of TNBC cases, compared to almost 25% in other breast cancer subtypes [166]. These mutations lead to the accumulation of oncogenic p53 in tumor cells, while the expression of wild-type (WT) p53 in normal cells is low. Since most of the mutations cause a single amino acid change in p53, tumor cell-derived p53 epitopes presented to T-cells commonly harbor WT sequences [167]. Thus, p53-targeted agents are attractive therapeutic options in a range of solid tumors. Accordingly, an MVA-based vaccine encoding full-length, WT human p53, named p53MVA, has been developed, which is able to deliver multiple immunogenic epitopes of p53 through various HLA molecules. In its first phase I trial of patients with refractory gastrointestinal malignancies, p53MVA was able to induce robust CD8+ responses, while being well-tolerated. However, no apparent clinical benefit was detected [168]. According to the existing data on potential synergy between viral vector vaccines and checkpoint inhibitors [169], and due to the fact that PD-1+ T-cells were frequently detected in the PBMCs of patients treated with single-agent p53MVA compared to healthy controls [168], the combination of this vaccine with pembrolizumab was tested in 11 patients with different types of solid tumors, of whom 7 were diagnosed with TNBC. In this phase I trial, up to three triweekly vaccine doses were administered along with pembrolizumab in patients with breast, pancreatic, hepatocellular, and head-and-neck cancers, and pembrolizumab monotherapy was continued every three weeks until disease progression occurred. Three of the eleven patients remained with stable disease for a maximum of 49 weeks, two of whom also showed p53-specific, CD8+ responses. Notably, one of the TNBC patients with cutaneous metastasis experienced complete regression for as long as six months after 9 weeks of treatment [59]. Five other TNBC patients were removed from the study at around week 10 due to disease progression, and the last TNBC patient had stable disease for 30 weeks. The adverse events observed in this trial were most probably attributed to pembrolizumab [60]. Further studies focusing on the selection of an appropriate patient population for this combination treatment through developing effective predictive biomarkers are warranted.

*Adenoviruses*

Adenoviruses are a family of DNA viruses which, as well as being highly immunogenic and efficient in gene delivery, are unable to integrate into the host genome. Adenoviral vectors are usually produced via mammalian packaging cell lines and administered directly as DNA vaccines [170].

NANT Cancer Vaccine (NCV)

A major hurdle in the immunotherapy of many solid tumors, including TNBC, is the immunosuppressive TME, which necessitates a multimodal approach to be overcome. Accordingly, an “orchestrated” strategy to achieve immunogenic cell death has been proposed by researchers at ImmunityBio: first, low-dose metronomic chemotherapy and stereotactic body radiotherapy (SBRT) induce tumor cell death thereby releasing TAAs; second, adenoviral and yeast vector-based cancer vaccines evoke anti-tumor T-cell immunity to the released TAAs; third, T-cell activity is further enhanced through the infusion of off-the-shelf, high-affinity natural killer (haNK) cells along with IL-15 superagonist N-803, and checkpoint inhibition. The cancer vaccines administered target a cascade of TAAs, including CEA, MUC1, brachyury, HER2, and RAS. In an early report of a phase Ib trial of the above-mentioned regimen in three patients with recurrent metastatic TNBC, two of the patients experienced a partial response (78% and 62% decrease) in the outpatient setting [64]. Patient accrual was continued thereafter, and, according to the most recent published report, nine TNBC patients have been collectively treated with this regimen. Four of the patients experienced grade ≥3 adverse events, of which two were haNK-associated. The disease control rate (including CR, PR, and SD) was 78%, and ORR was observed in five of the nine patients (56%), with two of the patients achieving CR (22%) [62]. Moreover, the levels of cell-free circulating RNA (cfRNA) of 18 relevant genes including PD-L1, CTLA-4, and HER2 were shown to be correlated with the response to treatment [171]. A randomized phase II study of NCV along with avelumab, chemotherapy, N-803, and haNK cells in phase II/III TNBC patients with the primary outcome of pCR has been submitted to clinicaltrials.gov, but has been withdrawn without any posted results (NCT03554109). The integrated approach of combining cancer vaccines with other chemo-, radio-, and immuno-therapeutic agents—rather than focusing on a single treatment approach—seems promising, and hence, further follow-up results of this trial are eagerly awaited.

PF-06936308

PF-06936308 is an investigational, adenovirus-based cancer vaccine developed by Pfizer. Encoding three non-disclosed TAAs, PF-06936308 has been investigated in patients with advanced/metastatic NSCLC and TNBC in a phase I trial (NCT03674827). The trial consists of two phases: the dose escalation phase, in which the safety and immunogenicity of increasing doses of the vaccine are evaluated, and the dose expansion phase, in which the preliminary efficacy of the vaccine is investigated in advanced/metastatic NSCLC patients. Although the first phase has been claimed to be completed, none of the results of the trial have been published. The current status of this trial is “terminated, due to the review of the asset within the sponsor’s portfolio.”

##### 3.3.2.2. Plasmid Vectors

Plasmids are closed circular DNA molecules which can serve as efficient vectors for DNA vaccines. Plasmids seem to be a safer alternative to viral vectors, and, unlike the latter, they do not trigger anti-vector immune responses [172]. Next-generation plasmid vectors have been developed, focusing on enhancing the immunogenicity of the plasmid DNA through the optimization of coding elements, or the inclusion of different molecular adjuvants in their structure [158].

Elenagen

Elenagen is a p62-encoding plasmid vaccine which, as well as inducing immune responses against p62, is known to alleviate chronic inflammation. p62 (sequestosome-1/SQSTM1), a protein involved in autophagy, apoptosis, tumor progression, and inflammatory signaling pathways, is a suitable target for cancer immunotherapy since it is overexpressed in most tumor cells but not in normal cells [173]. In the first in-human trial of Elenagen, 27 patients with advanced solid tumors (including 4 patients with TNBC) received at least 5 intramuscular injections of the vaccine. Elenagen proved to be safe without any dose-limiting toxicities, and the best overall response was stable disease in seven patients (of whom two were TNBC patients) for a maximum duration of 32 weeks. After finishing the course of vaccine therapy, conventional chemotherapy was administered to patients with once-stable or progressive disease. Of interest, chemotherapy regimens administered after Elenagen monotherapy caused more prolonged stable disease durations compared to first-line chemotherapy, suggesting the effect of Elenagen on restoring the sensitivity of cancer cells to chemotherapy, which can be partly attributed to its anti-inflammatory properties [66]. Considering this effect, the combination of Elenagen with chemotherapy could offer an effective treatment regimen, especially in breast cancer patients. Accordingly, the same team reported the case of a heavily-pretreated, chemo-resistant TNBC patient treated with the combination of weekly Elenagen and cyclophosphamide/methotrexate/fluorouracil (CMF) chemotherapy. The patient experienced a 33% partial tumor regression and 19 weeks of PFS [65]. Considering these positive results, further studies of such combinations on larger TNBC patient populations are eagerly awaited.

Polyepitope NeoAg DNA Vaccine

Researchers at the Washington University School of Medicine have developed a personalized DNA vaccine based on neoAgs from solid tumor specimens. The manufacturing process is similar to that of the personalized neoAg mRNA vaccines described above, and includes the steps of whole exome sequencing and bioinformatics-based computational analyses to choose the most suitable epitopes, which are then cloned into a mammalian expression vector. The vaccine has been studied in TNBC patients with persistent disease following neoadjuvant chemotherapy via IM electroporation, and assessed for safety and immunogenicity (NCT02348320). Meanwhile, according to preclinical data, while the vaccine was able to induce robust T-cell responses, it was alone unable to shrink the tumor in animal models. However, its combination with a PD-L1 inhibitor was effective in suppressing tumor growth [174]. Thus, the vaccine has been studied in combination with durvalumab in a phase II study of patients with small-cell lung (NCT04397003) and phase I study of patients with triple-negative breast (NCT03199040) cancers. The primary outcome in the latter is safety, and the secondary outcome is a specific immune response to the vaccine. While none of the results of the aforementioned trials have been published to date, an early report demonstrated the efficacy of the vaccine in eliciting neoAg-specific T-cell responses in a patient with a refractory pancreatic neuroendocrine tumor [174], suggesting its potential in the treatment of patients with advanced solid cancers, especially when combined with ICIs.

STEMVAC

STEMVAC is a multi-Ag plasmid DNA vaccine encoding Th1 epitopes of CD105, Yb-1, SOX2, CDH3, and MDM2: five important TAAs associated with cancer stem cells and the EMT process in breast cancer. Upon intradermal injection of this vaccine, transfected cells express the five TAAs, inducing specific memory and cytotoxic T-cell responses against the tumor. An active phase I, dose-escalation trial of STEMVAC combined with GM-CSF is evaluating the safety and induction of Th1 immune responses in 41 patients with stage III/IV HER2-negative BC [175]. Meanwhile, two separate phase II trials of STEMVAC in patients with early TNBC (NCT05455658) and metastatic NSCLC (NCT05242965) are running. In the former, 33 patients with up-to-stage III TNBC receive three monthly doses of the vaccine, followed by two booster shots in combination with GM-CSF in 3 and 9 months from the third dose. The primary outcome measure is the induction of the specific Th1 immune response to the five TAAs in different time frames after inoculation, and the secondary outcomes include safety and DFS. The study is anticipated to be completed by 2024.

## 4. Discussion

Although once perceived as a “cold” tumor type, there is now compelling evidence that TNBC is one of the most immunogenic subtypes of breast cancer. Accordingly, approaches to further “heat up” its TME are gaining popularity. Cancer vaccines, as novel active immunotherapeutic approaches, have started to find their way into TNBC treatment. While earlier studies focused on targeting the classical TAAs in TNBC through small peptide-based platforms, interest in personalized approaches of targeting individualized neoepitopes through cell- or nucleic acid-based vaccines is growing.

Cancer vaccines have demonstrated promising results in terms of safety in almost all clinical studies. However, many trials have failed to reveal clinically significant improvements in treatment responses. Several factors might have contributed to this observation. First, most studies of cancer vaccines with results for the TNBC subpopulation have focused on the breast cancer population as a whole, and a lack of trials with large populations of TNBC patients is obvious in most trials. Since breast cancer is considered to be a highly heterogenous cancer type with diverse tumor milieus, it is expected that different histological subtypes will respond quite differently to immunotherapeutic approaches, which is also perceivable from the results of some trials on the heterogenous population of breast cancer patients. Thus, it is important to stay focused on breast cancer subpopulations who are expected to better respond to cancer vaccines. Second, since most cancer vaccines under development for TNBC are in investigational phase I/II trials, the lack of randomized, multi-center trials hampers any clear conclusion about their actual benefit. Thus, further randomized phase III trials on vaccines with promising phase I/II results are anticipated. Third, when summarizing the results of different trials, it is important to keep in mind the homogeneity of the target populations in terms of the disease stage, background and prior treatment regimens, immune profiling, and co-treatments. One important point about cancer vaccines in general is that these active immunotherapeutic modalities are most effective within a relatively “healthy,” but not exhausted, tumor immune microenvironment. Therefore, patients with heavily pretreated tumors are unlikely to benefit from cancer vaccines, and it might be better to keep the focus on earlier stage patients. Since the included trials focused on very heterogenous populations of TNBC patients, we were unable to give a quantitative measure of the treatment effect of all studies. Hence, it is expected that, with the publication of the results of the many currently active trials, we can give a more comprehensive summary of the actual effect of the intervention. Fourth, it should be noted that breast cancer treatment is moving toward multi-modal treatment approaches. Thus, as a measure to enhance the potentials of vaccine therapy, evaluating its combination with chemo, radio, or immunotherapeutic approaches in future trials is warranted.

Despite all the pitfalls of the current studies, it should not be ignored that the area of vaccine therapy in TNBC is just in its infancy, but with very promising results so far. We expect that, with future larger trials focusing on homogenous TNBC populations with early-stage disease, the benefit of this treatment approach will stand out as a major therapeutic tool in the treatment of this deadly disease.

## Data Availability

No new data were created or analyzed in this study. Data sharing is not applicable to this article.

## Supplementary Materials

The following supporting information can be downloaded at: https://www.mdpi.com/article/10.3390/vaccines11010146/s1, File S1: Complete Search Strategy; Table S1: Quality assessment of randomized trials based on Modified Jadad Scale; Table S2: Quality assessment of non-randomized studies based on JBI Critical Appraisal Checklist.
