# Supplementary material for: Cancer Vaccines for Triple-Negative Breast Cancer: A Systematic Review
Source: Vaccines (Basel). 2023 Jan 9;11(1):146. doi: 10.3390/vaccines11010146 (PMC9866612; doi:10.3390/vaccines11010146)
Supplement: Supplementary file 1 [file vaccines-11-00146-s001.zip › vaccines-2076222-supplementary.pdf]

# Supplementary File S1- Complete search strategy.

## 1) Pubmed:

**Population:**

"Triple Negative Breast Neoplasms"[Mesh] OR TNBC[tiab] OR TNBCs[tiab] OR "triple receptor negative"[tiab] OR "triple negative"[tiab]

**Intervention:**

"Cancer Vaccines"[Mesh] OR vaccin* OR (immun*[tiab] AND adjuvant*[tiab]) OR immunoadjuvant*[tiab] OR (active[tiab] AND immun*[tiab]) OR immunis*[tiab] OR immuniz*[tiab]

## 2) Scopus:

**Population:**

TITLE-ABS-KEY ( "ER negative PR negative HER2 negative" ) OR TITLE-ABS-KEY ( "ER negative PR negative HER 2 negative" ) OR TITLE-ABS-KEY ( "TNBC*" ) OR TITLE-ABS-KEY ( "triple negative" ) OR TITLE-ABS-KEY ( "triple receptor* negative" )

**Intervention:**

TITLE-ABS-KEY ( vaccin* ) OR TITLE-ABS-KEY ( immuniz* ) OR TITLE-ABS-KEY ( immunis* ) OR TITLE-ABS-KEY ( immun* NEAR/1 adjuvant* ) OR TITLE-ABS-KEY ( immunoadjuvant* ) OR TITLE-ABS-KEY ( immun* NEAR/3 (active* OR cancer* OR anticancer*) )

## 3) Web of Science:

**Population:**

TS=("ER negative PR negative HER2 negative" OR "ER negative PR negative HER 2 negative" OR "TNBC*" OR "triple negative" OR "triple receptor* negative")

**Intervention:**

TS=(vaacin* OR immunis* OR immuniz* OR (immun* NEAR/1 adjuvant*) OR immunoadjuvant* OR ((active* OR cancer* OR anticancer*) NEAR/3 (immun* OR vaccin*)))

## 4) Embase:

**Population:**

'triple negative breast cancer'/exp OR 'tnbc' OR 'triple negative breast cancer' OR 'triple negative breast cancers' OR 'triple negative breast carcinoma' OR 'triple negative breast carcinomas' OR 'triple negative breast neoplasm' OR 'triple negative breast neoplasms' OR 'triple receptor negative breast cancer' OR 'triple receptor negative breast cancers' OR 'triple negative' OR 'triple receptor* negative' OR tnbc* OR 'er negative pr negative her 2 negative'

**Intervention:**

'cancer vaccine'/exp OR 'cancer vaccine' OR 'cancer vaccines' OR vaccin* OR immuniz* OR immunis* OR ’immunologic adjuvant’/de OR (immun* NEXT/1 adjuvant*) OR immunoadjuvant* OR ’active immunization’/de OR ’cancer immunization’/de OR vaccination/de OR ((active* OR cancer* OR anticancer*) NEAR/3 (immun*))

**<u>5) Cochrane CENTRAL:</u>**

**Population:**

"ER negative PR negative HER2 negative" OR "ER negative PR negative HER 2 negative" OR TNBC* OR "triple negative" OR "triple receptor negative"

**Intervention:**

vaccin* OR immuniz* OR immunis* OR (immun* NEXT/1 adjuvant*) OR immunoadjuvant* OR ((immun* OR vaccin*) NEAR/3 (active* OR cancer* OR anticancer*))

**<u>6) Google Scholar:</u>**

TNBC|“triple negative”|“triple receptor negative” “active|cancer immunization|immunotherapy”|vaccination

**<u>7) Clinicaltrials.gov:</u>**

**Population:**

Triple Negative Breast Neoplasms OR triple receptor negative OR triple negative OR TNBC

**Intervention:**

Vaccin OR immunis OR immuniz OR immune adjuvant OR immunoadjuvant

## Supplementary Table S1- Quality assessment of randomized trials based on Modified Jadad Scale.

| Study | Was the study described as randomized | Was the method of randomization appropriate? | Was the study described as blinding? | Was the method of blinding appropriate? | Was there a description of withdrawals and dropouts? | Was there a clear description of the inclusion/exclusion criteria? | Was the method used to assess adverse effects described? | Were the methods of statistical analysis described? | Total Score |
|---|---|---|---|---|---|---|---|---|---|
| Mittendorf et al. 2016 [25] | 1 | 1 | 0.5 | 1 | 1 | 1 | 1 | 1 | 7.5 |
| Brown et al. 2020 [26] | 1 | 0 | 0.5 | 0 | 1 | 1 | 1 | 1 | 5.5 |
| Chick et al. 2021 [33] | 1 | 1 | 0.5 | 1 | 0 | 1 | 1 | 1 | 6.5 |
| Clifton et al. 2020 [34] | 1 | 1 | 0.5 | 1 | 1 | 1 | 1 | 1 | 7.5 |
| Singer et al. 2020 [39] | 1 | 0 | 0 | 0 | 0 | 1 | 1 | 1 | 4 |
| Huang et al. 2020 [40] | 1 | 1 | 1 | 1 | 1 | 1 | 1 | 1 | 8 |

## Supplementary Table S2- Quality assessment of non-randomized studies based on JBI Critical Appraisal Checklist.

Q1, Is it clear in the study what is the 'cause' and what is the 'effect'; Q2, Were the participants included in any comparisons similar?; Q3, Were the participants included in any comparisons receiving similar treatment/care, other than the exposure or intervention of interest?; Q4, Was there a control group?; Q5, Were there multiple measurements of the outcome both pre and post the intervention/exposure?; Q6, Was follow up complete and if not, were differences between groups in terms of their follow up adequately described and analyzed?; Q7, Were the outcomes of participants included in any comparisons measured in the same way?; Q8, Were outcomes measured in a reliable way?; Q9, Was appropriate statistical analysis used?

| Study | Q1 | Q2 | Q3 | Q4 | Q5 | Q6 | Q7 | Q8 | Q9 | % Yes |
|---|---|---|---|---|---|---|---|---|---|---|
| Takahashi et al. 2014 [45] | Yes | No | No | No | Yes | Yes | Yes | Yes | Yes | 66.67 |
| Toh et al. 2020 [49] | Yes | Yes | No | No | Yes | Yes | Yes | Yes | Yes | 77.78 |
| Santisteban et al. 2021 [52] | Yes | Yes | Yes | Yes | Yes | Yes | Yes | Yes | Yes | 100 |
| Chung et al. 2019 [60] | Yes | No | No | No | Yes | Yes | Yes | Yes | No | 55.56 |
| Ponomarenko et al. 2017 [66] | Yes | No | No | No | Yes | Yes | Yes | Yes | No | 55.56 |
